# Consequences of Maternal Essential Fatty Acid and Conjugated Linoleic Acid Supplementation on the Development of Calf Muscle and Adipose Tissue

**DOI:** 10.3390/ani10091598

**Published:** 2020-09-08

**Authors:** Nina Dahl, Elke Albrecht, Dirk Dannenberger, Katrin L. Uken, Harald M. Hammon, Steffen Maak

**Affiliations:** 1 Institute of Muscle Biology and Growth, Leibniz Institute for Farm Animal Biology (FBN), 18196 Dummerstorf, Germany; dahl@fbn-dummerstorf.de (N.D.); dannenberger@fbn-dummerstorf.de (D.D.); maak@fbn-dummerstorf.de (S.M.); 2 Institute of Nutritional Physiology “Oskar Kellner”, Leibniz Institute for Farm Animal Biology (FBN), 18196 Dummerstorf, Germany; uken@fbn-dummerstorf.de (K.L.U.); hammon@fbn-dummerstorf.de (H.M.H.)

**Keywords:** adipocyte, gas chromatography, histology, maternal transfer, muscle structure, myosin heavy chain isoforms, PUFA

## Abstract

**Simple Summary:**

Cows in modern dairy production systems are fed with silage and concentrate-based diets, resulting in a low essential fatty acid and conjugated linoleic acid status in body tissues and milk. During the late pregnancy and early postnatal period, a reduced maternal fatty acid supply might negatively affect calf development. This study investigated the effects of a maternal re-substitution with essential fatty acids and conjugated linoleic acids on muscle and fat tissue development of neonatal calves. The maternally-supplemented fatty acids were found to be elevated in calf skeletal muscle and fat tissues, but no short-term effects on tissue structure were observed. Nevertheless, the possibility to change offspring fatty acid status via maternal nutrition has been confirmed that could influence offspring development and health in the long-term.

**Abstract:**

Common silage and concentrate-based diets in dairy and beef production may deliver insufficient amounts of essential fatty acids (EFA), thereby also reducing conjugated linoleic acids (CLA) in body tissues and milk. An impaired maternal EFA and CLA supply can have an important impact on calf postnatal development. The current study investigates how maternal supplementation with EFA and CLA affects muscle and adipose tissue development in neonatal calves. Holstein cows (n = 40) were abomasaly supplemented with coconut oil (control), CLA or EFA, or both combined during the transition period. Calves were fed their dam’s colostrum until slaughter at day 5 of life. Fatty acid composition and tissue morphology were analyzed. In muscle and adipose tissues, EFA, CLA, and metabolites were elevated, indicating the effective transfer of maternally-supplemented FA to the offspring. Muscle fiber types, fiber nuclei, myosin heavy chain isoform distribution, capillarization, and fat cell size of intramuscular and other adipose tissues did not differ among groups. The results confirm that maternal nutrition during the transition period can alter the FA composition of the calf tissues. This could influence the offspring’s development and health in the long-term, even though only minor effects were observed in the neonatal calves’ tissue morphology.

## 1. Introduction

Common diets in dairy and beef production are mainly based on corn silage and concentrates to meet the energy demand of high-yielding dairy cows and to improve growth efficiency of beef cattle [1,2,3]. Those diets contain low amounts of essential fatty acids (EFA), especially α-linolenic acid (C18:3 *n*-3, ALA). The organism cannot synthesize EFA endogenously [4] and relies on uptake via the diet. Linoleic acid is the most abundant dietary *n*-6 polyunsaturated fatty acid (PUFA) and it can be found in vegetable oils, such as corn, safflower, and linseed oil, whereas ALA appears mainly in chloroplasts of green vegetables and grass [5]. For the bovine, fresh grass used to be the main EFA source. However, all corn-based diets may deliver insufficient amounts of EFA to the bovine. Additionally, rapid ruminal biohydrogenation reduces the amounts of EFA available for intestinal absorption even more [6,7].

Linoleic acid and ALA affect many physiological processes. They are incorporated into phospholipids, serving both structural and signaling purposes [8,9]. Essential fatty acids are precursor molecules of other unsaturated fatty acids with distinct metabolic functions, like eicosapentaenoic acid (EPA), *n*-3 docosapentaenoic acid (*n*-3 DPA) and docosahexaenoic acid (DHA) [10]. In addition, LA and ALA are the main precursors for conjugated linoleic acid (CLA) formation [7,11,12]. Conjugated linoleic acids (C18:2) are synthesized from EFA in the rumen; or from the LA metabolite vaccenic acid (C18:1 *trans*-11) in the mammary gland [13]. An EFA reduced diet, therefore, results in less rumen production of CLA and *trans*-fatty acids, leading to reduced CLA in body tissues and milk [14,15].

Nutrition of the cow during gestation has a profound impact on fetal muscle development [16]. Until birth, the formation of muscle fibers in the fetus is completed, but myogenic cells continue to proliferate and fuse with existing muscle fibers to enable muscle fiber hypertrophy [16]. On the other hand, adipogenesis in the muscle is initiated and major formation of adipocytes in different adipose tissues occurs during late gestation and early postnatal life [16]. Thus, maternal nutrition during this period may affect adipocyte hyperplasia and with that the overall adipose tissue development, including intramuscular clusters of adipocytes [17]. Nutrients, also supplemented FA, are transported from the cow to the fetus and the neonate via placenta and colostrum, respectively [18]. Due to the importance of neonatal EFA and CLA supply for body functions, such as cell integrity, immune response, and energy metabolism, the EFA and CLA status of the mother has an important impact on health and development in the neonate [19,20,21].

In calves supplemented with EFA, higher growth rate and anabolic rates were observed [22]. Additionally, cell culture experiments revealed increased calf skeletal muscle cell differentiation upon supplementation with ALA and *cis*-9, *trans*-11 CLA [23,24]. Adipocyte numbers were increased upon incubation of intramuscular and subcutaneous tissue explants from young steers, with ALA or *trans*-10, *cis*-12 CLA [25]. Based on this, we hypothesized that maternal supplementation with EFA and CLA during the late prenatal and early postnatal period can augment the development of skeletal muscle and adipose tissue in dairy calves. This study assessed how a maternal supplementation with EFA and CLA, in comparison to saturated fatty acids (SFA), affected FA composition, and skeletal muscle and adipose tissue cellularity in calves at day 5 after birth.

## 2. Materials and Methods

### 2.1. Animals

The animal experiment was divided into five time blocks with eight animals each and a periodical difference of three months. Animal care and tissue collection procedures followed the guidelines of the German Law of Animal Protection. The experimental protocol was approved by the Animal Protection Board of the Leibniz Institute for Farm Animal Biology as well as by the Animal Care Committee of the State Mecklenburg-Western Pomerania, Germany (State Office for Agriculture, Food Safety and Fishery; LALLF M-V/TSD/7221.3-1-052/15).

Forty pregnant Holstein cows were adapted to a standard diet for high yielding dairy cows with reduced content of EFA, starting 16 weeks before the estimated parturition. All cows were fed a corn silage-based total mixed ration (starch: 246 g/kg dry matter (DM), nRP: 159 g/kg DM, NE_L_: 6.92 MJ/kg DM, fat content: 21 g/kg DM, *n*-6 FA: 10.83 g/kg DM, *n*-3 FA: 1.0 g/kg DM, *n*-3:*n*-6 ratio: 0.09), as described by Vogel et al. [26]. From 63 days before estimated parturition on, the control group (CON) was supplemented twice a day with coconut oil (90% saturated FA) at 38 g/day during dry period and 76 g/day during lactation period. The EFA group was supplemented with linseed oil (LO) and safflower oil (SO) (50% *n*-3 FA, 15% *n*-6 FA) at 41 g/day during dry period (LO: 39 g/day, SO: 2 g/day) and 82 g/day during lactation period (LO: 78 g/day, SO: 4 g/day) [26]. The CLA group was supplemented with Lutalin® (35% MUFA, 15% SFA, 5% PUFA; 25% *cis*-9, *trans*-11 CLA, 25% *trans*-10, *cis*-12 CLA; BASF, Ludwigshafen, Germany) at 19 g/day during dry period and 38 g/day during lactation period. The EFA + CLA group received the combined rations of the EFA and CLA groups (LO, SO and Lutalin®) at 60 g/day during dry period and 120 g/day during lactation period. Supplementation was given via abomasal fistula (for details, see [26]).

After birth, calves were kept under identical conditions and were fed with their dam’s colostrum (12% of body weight) until slaughter at day 5 of life. Samples of 36 calves were finally harvested, due to stillbirth losses and technical reasons, of the groups CON (three female, five male), EFA (five female, four male), CLA (seven female, one male) and EFA + CLA (seven female, four male). Samples of musculus longissimus dorsi (MLD), musculus semitendinosus (MST), subcutaneous fat (SCF), intermuscular fat (INF) and kidney fat (KF) were taken within 30 min after slaughter, frozen and stored at −20 °C (FA analysis, muscle cross-sectional area analysis) or frozen in liquid nitrogen and stored at −70 °C (all others).

### 2.2. Lipid Extraction and Fatty Acid Analysis

Frozen muscle and adipose tissue samples were homogenized and nonadecanoic acid (C19:0) was added as an internal standard. Total tissue lipids were extracted in duplicate using chloroform/methanol (2:1, *v*/*v*) and the Ultra Turrax T25 (IKA, Staufen, Germany) at three times 15 s, 15,777× *g* and room temperature (RT). The detailed workflow of lipid extraction has been described recently by Kalbe et al. [27]. The preparation of fatty acid methyl esters (FAME) was performed in two reaction steps, first treatment with 0.5 M sodium methoxide in methanol followed by treatment with 14% boron trifluoride (BF_3_) in methanol. After FAME extraction with *n*-hexane, the extracts were stored at −18 °C until used for gas chromatography (GC) analysis. The fatty acid analysis of the tissue lipids was performed using capillary GC with a CP-Sil 88 CB column (100 m × 0.25 mm; Agilent, Santa Clara, CA, USA), that was installed in a PerkinElmer gas chromatograph CLARUS 680 with a flame ionisation detector and split injection (PerkinElmer Instruments, Shelton, CT, USA). For the calibration procedure the reference standard mixture ‘Sigma FAME’ (Sigma-Aldrich, Deisenhofen, Germany), the methyl ester of C18:1 *cis*-11, C22:5 *n*-3 and C18:2 *cis*-9, *trans*-11 (Matreya, State College, PA, USA), C22:4 *n*-6 (Sigma-Aldrich, Deisenhofen, Germany) and C18:4 *n*-3 (Larodan, Limhamn, Sweden) were used. The five-point calibration of single fatty acids ranged between 16 and 415 µg/mL and was checked after GC analysis of five samples. The detailed GC conditions were recently described by Dannenberger et al. [28].

### 2.3. Muscle Cross-Sectional Area Measurement

One centimeter thick muscle slices of MLD were taken at the twelfth rib and muscle slices of MST were taken at the thickest part of the muscle belly. Muscle slices were placed on a pad with a scale, and images of both transverse sides were taken from top view, with a Nikon Coolpix 8700 camera (Nikon, Düsseldorf, Germany).

With Cell^D analysis software (OSIS, Münster, Germany), images were calibrated to the scale. The outline of the muscle was drawn with the interpolating polygon function of the interactive measurement module of Cell^D, and the area of both sides was determined. The mean total muscle area per sample was calculated from the area measured at each side of one slice. The apparent total muscle fiber number per muscle was extrapolated from the number of muscle fibers per mm^2^, determined during muscle fiber typing (see Section 2.4, below).

### 2.4. Histology

Adipose tissue samples were cut into serial sections, 30 µm thick, using a cryostat microtome (CM3050 S, Leica, Bensheim, Germany). Muscle samples were cut 12 µm thick and the serial sections were stained with either Oil Red O (Chroma Gesellschaft, Münster, Germany), hematoxylin/eosin (H/E, hematoxylin: Dako, Glostrup, DK; eosin: Chroma Gesellschaft, Münster, Germany), or were used for myofibrillar ATPase staining and capillary staining (alkaline phosphatase reaction) with standard procedures.

In adipose tissue sections, cross-sectional area (µm^2^) and diameter (µm) of at least 300 fat cells in each tissue were measured in ten randomly selected regions of H/E-stained sections. For this procedure, the interpolating polygon function of the interactive measurement module of Cell^D analysis software (OSIS, Münster, Germany) was applied. Images were taken using the 40× objective at an Olympus BX43 microscope (Olympus, Hamburg, Germany), equipped with an UC30 color camera.

The same equipment was used to determine the adipocyte lipid droplet area percentage in Oil Red O stained muscle sections. At least three serial sections per sample (total area > 44 mm^2^) were analyzed. From each serial section, the total section area was measured using the 1.25× objective and the interactive measurement module of Cell^D. All individual adipocyte lipid areas were measured using the 20× objective and a macro program as follows: The green channel of the original image was extracted, the contrast enhanced, the threshold defined for detection of lipid areas, and the area of lipids measured. The percentage of total lipid area per slice was calculated dividing the sum of total lipid area from all serial sections of a sample by the area sum of the serial sections multiplied by 100.

To identify the muscle fiber types as slow twitch, fast twitch, or intermediate [29], muscle sections were stained for myofibrillar ATPase, according to Szentkuti and Eggers [30] with alkaline preincubation. For each sample at least 300 fibers, in three randomly selected regions of the same slice, were analyzed with the muscle fiber measurement module (MAS, Freiburg, Germany) of the Cell^D software, as described in detail by Albrecht et al. [31]. Muscle fiber size, nuclei, and fiber type composition were analyzed.

Capillarization was determined in eosin and alkaline phosphatase stained sections [32]. Three representative images were taken with the 12.5× objective, and capillary size and density were analyzed with a macro program for capillarization. Additionally, muscle fibers were counted in one representative part of each image at higher magnification. The procedure has been described by Zitnan et al. [33]. The analysis included determination of muscle fiber number per area unit, capillary size, capillary number per mm^2^, number of muscle fibers per capillary, area percentage of capillaries, and distance between capillaries.

### 2.5. Immunohistochemistry

Longissimus muscle tissue sections were fixated with 4% paraformaldehyde (Carl Roth, Karlsruhe, Germany) for 15 min. Slides were then washed twice with PBS for 10 min and permeabilized using PBS-Triton (PBST, 0.1% Triton X-100 (Sigma-Aldrich, Munich, Germany) in PBS). After blocking with 10% normal goat serum for 15 min, the sections were incubated with the primary antibody mix (2% normal goat serum, 1:200 anti-MYH7 mouse monoclonal antibody (Abcam, Cambridge, UK), 1:300 anti-MYH2 rabbit polyclonal antibody (Abcam) in PBST) for 1 h at RT in a humidified chamber. After washing three times with PBST, slides were incubated with the secondary antibody mix (Alexa 488 goat anti-rabbit IgG and Alexa 594 goat anti-mouse IgG (both: Thermo Fisher Scientific, Schwerte, Germany), 1:1000 in PBST) for 45 min at RT in the dark in a humified chamber. Nuclei were counterstained with Hoechst 33258 (1 µg/mL in PBS, Sigma-Aldrich, Munich, Germany) for 5 min, slides were washed twice with PBS and distilled water and finally mounted with Pro Long Diamond Antifade Mountant (Thermo Fisher Scientific, Schwerte, Germany).

Immunofluorescence was detected on a Nikon Microphot SA fluorescence microscope (Nikon, Duesseldorf, Germany) equipped with a CC-12 high resolution color camera (OSIS, Münster, Germany) and Cell^F imaging software (OSIS, Münster, Germany). With the 10× objective, at least three images per sample were taken and analyzed for the area percentage of MYH2 and MYH7 expressing fibers, respectively. Steps of contrast enhancement, extraction of red or green channel, for MYH7 and MYH2 fibers, respectively, threshold setting, and phase analysis were part of the macro program. With the program, areas occupied by each fiber type were determined. To analyze the hybrid fibers, expressing both MYH2 and MYH7, at least three images per sample were taken using the 20× objective in both red and green channel. Within secondary muscle fiber bundles, hybrid fibers were counted. The fluorescent signal of hybrid fibers in each channel was compared to the single expressing fibers as equally or less strong. For data analysis, the hybrid fibers were distributed into three groups, depending on the signal intensity in both channels.

### 2.6. Protein Extraction and Western Blotting

Muscle samples were homogenized with the Xiril Dispomix (Xiril, Hombrechtikon, Switzerland) in CelLytic MT Lysis Reagent and Protease Inhibitor Cocktail (both Sigma-Aldrich, Munich, Germany) to extract total protein according to manufacturer’s instructions. Tissue lysates were centrifuged at 20,817× *g* for 15 min at RT and the supernatants were transferred into new tubes. The protein concentration was measured via a Nanodrop spectrometer (ND-1000, Peqlab, Erlangen, Germany). Protein samples were diluted to 1 mg/mL and 2 µg were separated via sodium dodecyl sulfate polyacrylamide gel electrophoresis (SDS-PAGE). The Smart Protein Layers (SPL) Red Kit (PR926, NH DyeAGNOSTICS, Halle, Germany) was used for normalization and quantification of protein abundances. Proteins were denatured at 95 °C for 5 min and separated on 4–15% Criterion TGX Stain-Free Precast gels (Bio-Rad, Munich, Germany) using 0.1% SDS (*v*/*v*) in 1x Towbin buffer (25 mM Tris, 192 mM Glycine in Aqua dest.) at 175–200 V. Proteins were blotted on a polyvinylidene difluoride membrane (Trans-Blot Turbo RTA Mini PVDF Transfer Kit, Bio-Rad, Munich, Germany) with a semi dry blotter (Trans-Blot, Bio-Rad, Munich, Germany). All antibody incubations were done using the iBind Flex Solution Kit (Thermo Fisher Scientific, Schwerte, Germany). Primary antibodies were all purchased from Abcam (Cambridge, UK, anti-MYH1 chicken polyclonal antibody; anti-MYH2 rabbit monoclonal antibody; anti-MYH7 mouse monoclonal antibody). The MYH1 was discontinued by Abcam and replaced with a rabbit polyclonal anti-MYH1 antibody (Elabscience, Wuhan, China) for measurements in MST. Secondary antibody for MYH1 in MLD was purchased from Abcam (rabbit anti-chicken IgY H&L); Rabbit TrueBlot (anti-rabbit IgG HRP, Rockland Immunochemicals, Limerick, PA, USA) was used on MYH1 in MST, and on MYH2 in both muscles. Anti-MYH7 antibody was detected with Alexa Fluor 488 goat anti-mouse IgG (H+L) (Thermo Fisher Scientific, Schwerte, Germany).

Fluorescence of calibrators and total protein (for normalization), and chemiluminescence of target proteins were recorded with the Chemocam HR-16 imager system (Intas Science Imaging Instruments GmbH, Göttingen, Germany) and quantified using LabImage software (Kapelan Bio-Imaging GmbH, Leipzig, Germany).

### 2.7. Statistical Analysis

Data were analyzed with the MIXED model of SAS statistical software (SAS 9.4, SAS Institute, Cary, NC, USA). Fixed effects were the supplemented FA (EFA, CLA) at levels “yes” (if supplemented) or “no” (not supplemented) and their interaction, as well as calf sex and interaction with the supplemented FA. Maternal supplementation blocks were included as random factors. Gestation length (days) was included as a covariate in the model, but was removed where it has no significant effect (*p* > 0.05). Denominator degrees of freedom were calculated by Kenward-Roger estimation. Least Square Means (LSMs) were compared with the PDIFF statement and adjusted using the Tukey-Kramer post-hoc test. Data are depicted as LSM ± Standard Error of LSM (SE_LSM_). All correlated data was tested for normality distribution with the Shapiro-Wilk test via the UNIVARIATE procedure. The Spearman correlation was analyzed using the CORR procedure of SAS. The skewness factors of fat cell size histograms were analyzed with the MEANS procedure.

## 3. Results

### 3.1. Maternal FA Supplementation Changed FA Concentrations of the Calf Muscle Tissues

Maternal FA supplementation reached the skeletal muscle tissue of the calves and changed the FA profile in both muscles similarly (details in Supplementary Tables S1 and S2). In MLD (Table 1), *cis*-9, *trans*-11 CLA was enriched in samples of calves from CLA supplemented dams (*p* < 0.01). Additionally, ALA and longer-chain *n*-3 PUFAs were enriched in samples of calves from EFA supplemented dams (*p* < 0.05). Linoleic acid, C18:3 *n*-6 and C20:3 *n*-6 remained unchanged in EFA supplemented animals (*p* > 0.05). C20:4 *n*-6 (ARA) was decreased in the EFA group, compared to the CLA supplementation group (*p* = 0.032); while *n*-6 PUFAs C22:4 *n*-6 (ADA) and C22:5 *n*-6 were decreased in both EFA and EFA + CLA animals, compared to the CON and CLA supplementation groups (*p* < 0.05). Overall, *n*-3 PUFAs were enriched in EFA supplemented animals (*p* < 0.001), while *n*-6 PUFAs were unchanged upon supplementation (*p* > 0.05). Total PUFAs were unchanged by supplementation (*p* > 0.05) in MLD, while in MST (Supplementary Table S2), total PUFAs were elevated in the EFA + CLA group, compared to CON (*p* = 0.005). In MLD, but not in MST, C18:1 *c*-9 (OA) was lowered in the two EFA supplementation groups, compared to CON group (*p* < 0.05). The CLA precursor C18:1 *t*-11 (VA) was unchanged upon supplementation (*p* > 0.05) in MLD, but in MST, it was elevated in the EFA + CLA group, compared to CON and EFA (*p* < 0.05). Total monounsaturated FA (MUFA) concentration in MLD was increased in the CLA group, compared to EFA and EFA + CLA groups (*p* < 0.05). The saturated FA C16:0 (PA) remained unchanged by maternal supplementation in both muscles whereas C18:0 (SA) was elevated in the MST of EFA + CLA group, compared to CON and EFA supplementation group calves (*p* < 0.05). The overall fat percentage remained unchanged in both muscles (*p* > 0.05).

### 3.2. Maternal FA Supplementation Changed FA Composition of the Calf Adipose Tissues

At five days of life, SCF and INF were differently developed among animals as indicated by the range of overall fat percentage (SCF: 3.8 to 42.1% of tissue weight, INF: 0.81 to 45.33% of tissue weight). In SCF, the LSM of total fat content (g/100 g tissue) did not differ between the groups, with an overall LSM of 18.17 ± 1.54 (LSM ± SE_LSM_). Therefore, the fatty acid composition (percentage of total fatty acids) instead of the absolute fatty acid concentration of SCF (as representative for both fat depots) is presented in Table 2 (for INF see Supplementary Table S4). In concordance with the muscle data, *cis*-9, *trans*-11 CLA was enriched in the SCF samples of calves from CLA supplemented dams (*p* < 0.05). In INF, CLA was unchanged by CLA supplementation, but lower in the EFA group, compared to the EFA + CLA group (*p* = 0.029). In the SCF, ALA, EPA and *n*-3 DPA were enriched in the samples of calves from EFA supplemented dams (*p* < 0.05), while in INF only ALA was higher in both EFA supplementation groups (*p* < 0.05). In the SCF, C18:3 *n*-6 was enriched in the CLA group (*p* < 0.001); and ADA was lowered in the EFA + CLA supplementation group, compared to CON (*p* = 0.04). Overall, in SCF, *n*-3 PUFAs were enriched in EFA supplemented animals (*p* < 0.01), while in INF (Supplementary Table S4) *n*-3 PUFAs were only enriched in the EFA + CLA group, compared to the CLA group (*p* = 0.044). In both tissues, *n*-6 PUFAs and total PUFAs were unchanged upon supplementation (*p* > 0.05). Oleic acid, VA and total MUFA percentage, PA, SFA percentage and total fat content were unaffected by supplementation (*p* > 0.05). In both SCF and INF, SA was elevated in the EFA + CLA supplementation groups compared to CON (*p* < 0.05).

Kidney fat was analyzed as representative for a more developed internal adipose tissue. Fatty acid concentrations (mg/100 g tissue) are shown in Table 3. *Cis*-9, *trans*-11 CLA was enriched in the CLA supplementation groups, compared to the EFA group (*p* < 0.05). As expected, ALA and longer-chain *n*-3 PUFAs were enriched in the samples of calves from EFA supplemented dams (*p* < 0.05). Only C18:4 *n*-3 (SDA) was decreased in the EFA + CLA group, compared to the control (*p* = 0.014). Concentrations of LA and longer-chain *n*-6 PUFAs were unchanged (*p* > 0.05), or below the quantification limit of the method (< 0.05 mg/100g tissue) (C22:5 *n*-6). *N*-3 PUFAs were enriched in the EFA supplementation groups (*p* < 0.01), whereas *n*-6 PUFAs, OA, *trans*-11 VA, PA, SA, total SFAs, and total fat percentage were similar in all groups (*p* > 0.05).

### 3.3. Muscle Fiber Type Composition Was Unchanged by Maternal FA Supplementation

**Figure 1:**
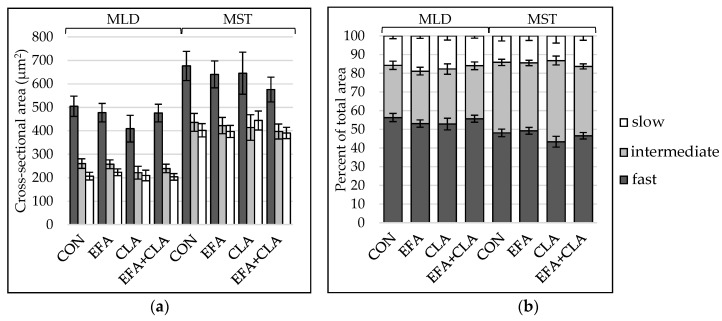
Muscle fiber type distribution in longissimus (MLD) and semitendinosus muscle (MST) of calves of four maternal supplementation groups: Control group (CON, n = 8), essential fatty acids (EFA, n = 9), conjugated linoleic acids (CLA, n = 8), EFA + CLA (n = 11). (**a**) Muscle fiber cross-sectional area (µm^2^). (**b**) Area percentage per fiber type. Data are shown as LSM ± SE_LSM_.

To determine, if the maternal FA supplementation had led to changes in total muscle fiber number, the muscle fiber number per mm^2^ was extrapolated to the muscle cross-sectional area. The apparent total muscle fiber number was higher in MLD (LSM ± SE_LSM_ (×10^6^): 6.06 ± 0.18) than in MST (LSM ± SE_LSM_ (×10^6^): 3.68 ± 0.13), but there was no difference between the groups (*p* > 0.05). To elucidate whether the FA supplementation had affected the muscle fiber composition, the contraction type was determined using ATPase staining with alkaline preincubation. Muscle fibers of all three types were larger in MST than in MLD (Figure 1a), and the fiber type composition differed between the muscles (Figure 1b), with less fast and more intermediate fibers in MST. Group differences were not observed (*p* > 0.05). The number of nuclei per muscle fiber was higher in MLD than in MST without significant differences among the groups (*p* > 0.05, Supplemental Figure S1).

**Figure 2:**
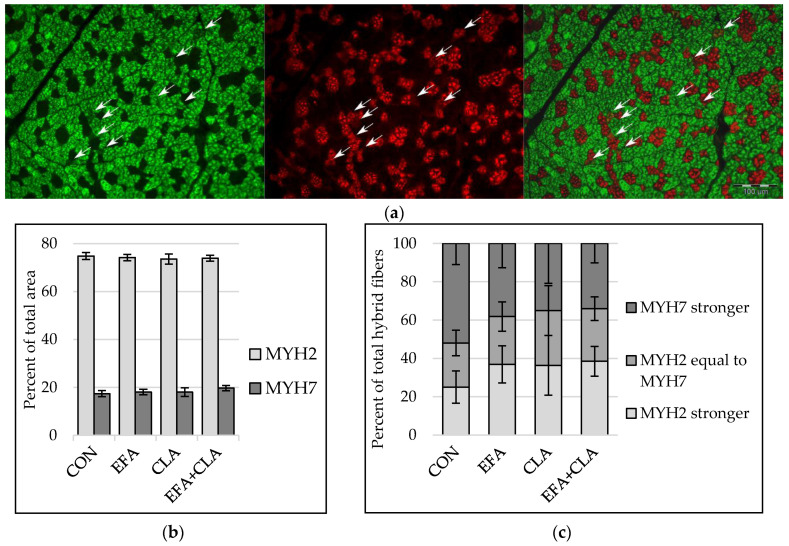
Immunohistochemical detection of myosin heavy chain isoform 2 (MYH2) and isoform 7 (MYH7) in longissimus muscle of calves of four maternal supplementation groups: Control group (CON, n = 8), essential fatty acids (EFA, n = 9), conjugated linoleic acids (CLA, n = 8), EFA + CLA (n = 11). (**a**) Representative immunofluorescence images of muscle fiber cross-sections stained for MYH2 (left, green), MYH7 (middle, red) and merged (right). Arrows indicate hybrid fibers. Scale bar = 100 µm. (**b**) Area percentage of MYH2 and MYH7 expressing fibers. (**c**) Percentage of total hybrid fibers for hybrid fiber groups, formed according to signal intensity. Data are shown as LSM ± SE_LSM_.

To elucidate whether the FA supplementation affected the transition of muscle fibers from one type to another (slow to fast or vice versa), the slow and fast isoforms of myosin heavy chains (MYH) were determined with immunohistochemistry, with a special focus on fibers expressing both isoforms (“hybrid fibers”) (Figure 2a). The area percentage of MYH2 correlated with the area percentage of fast and intermediate fibers (Spearman correlation coefficient: 0.55) and the area percentage of MYH7 with that of slow fibers (Spearman correlation coefficient: 0.72, *p* < 0.001). The area percentage of muscle fibers expressing either MYH2 or MYH7 was similar in all groups (*p* > 0.05, Figure 2b). Fibers expressing both MYH2 and MYH7 were divided into three groups according to the stronger fluorescent signal. The apparent increase of hybrid fibers with a stronger MYH2 signal and reduced number of fibers with a stronger MYH7 signal in all supplemented groups compared to the control group was not statistically significant (*p* > 0.05, Figure 2c).

**Figure 3:**
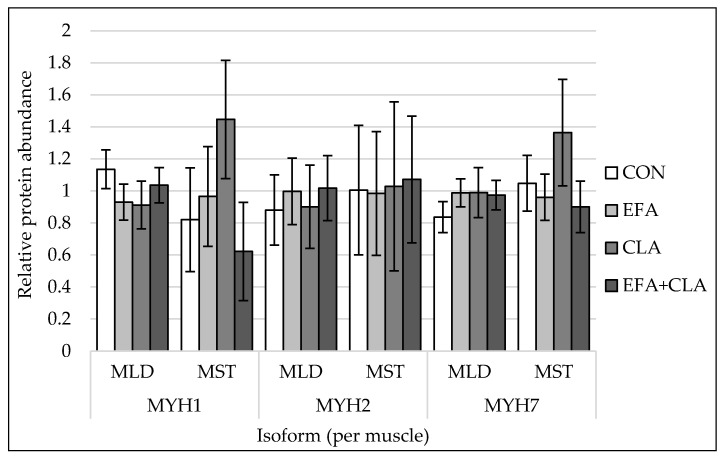
Protein quantification of myosin heavy chain isoform 1 (MYH1), isoform 2 (MYH2) and isoform 7 (MYH7) in longissimus (MLD) and semitendinosus muscle (MST) of calves of four maternal supplementation groups: Control group (CON, n = 8 (MLD), n = 5 (MST)), essential fatty acids (EFA, n = 9 (MLD), n = 7 (MST)), conjugated linoleic acids (CLA, n = 8 (MLD), n = 5 (MST)), EFA + CLA (n = 11 (MLD), n = 7 (MST)). Relative protein abundance, normalized against total protein amount per sample. For representative blots of target protein and total protein, see Supplemental Figure S2. Data are shown as LSM ± SE_LSM_.

The protein abundances of myosin heavy chain isoforms 1, 2, 7, and 8 were quantified by western blots. For representative blot pictures of target protein and total protein, see Supplemental Figure S2. There were no differences among the supplementation groups observed (*p* > 0.05, Figure 3) for any of the investigated MYH isoforms.

### 3.4. Muscle Capillarization Was Not Affected by Maternal FA Supplementation

**Figure 4:**
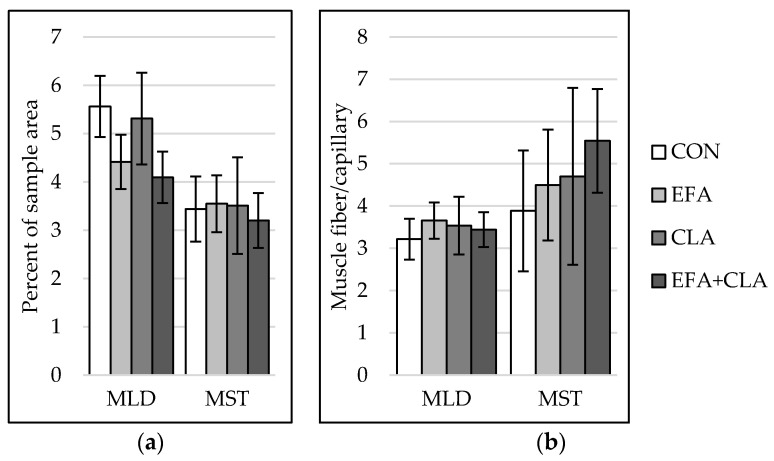
Capillarization in longissimus (MLD) and semitendinosus muscle (MST) of calves of four maternal supplementation groups: Control group (CON, n = 8), essential fatty acids (EFA, n = 9), conjugated linoleic acids (CLA, n = 8), EFA + CLA (n = 11). (**a**) Percent of sample area. (**b**) Number of muscle fibers per capillary. Data are shown as LSM ± SE_LSM_.

The capillarization was analyzed to investigate the influence of FA supplementation on blood and nutrient supply within the muscles. In both muscles, capillarization (Figure 4a) and muscle fiber-to-capillary ratio (Figure 4b) did not differ between the groups (*p* > 0.05).

### 3.5. No Effects of Maternal FA Supplementation on Intramuscular Fat Deposition

**Figure 5:**
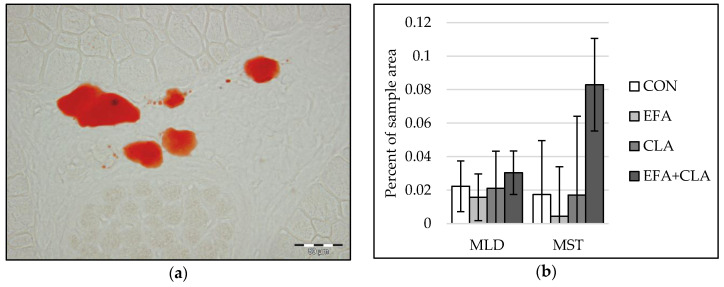
Intramuscular fat in longissimus (MLD) and semitendinosus muscle (MST) of calves of four maternal supplementation groups: Control group (CON, n = 8), essential fatty acids (EFA, n = 9), conjugated linoleic acids (CLA, n = 8), EFA + CLA (n = 11). (**a**) Representative picture of adipocyte lipid droplets (red) in Oil Red O staining. Scale bar = 50 µm. (**b**) Percentage of lipid droplet area of the total sample area. Data are shown as LSM ± SE_LSM_.

Only a few adipocytes were observed in muscle tissue of calves at five days of age. Therefore, instead of individual adipocyte sizes, we determined the area of intramuscular lipids within developing adipocytes in Oil Red O stained muscle cross-sections with image analysis (Figure 5a). The values varied largely among all calves. Thus, differences in the intramuscular adipocyte lipid droplet area were not observed among the groups (*p* > 0.05, Figure 5b).

### 3.6. Maternal FA Supplementation Changed the Fat Cell Size Distribution in Different Depots

**Figure 6:**
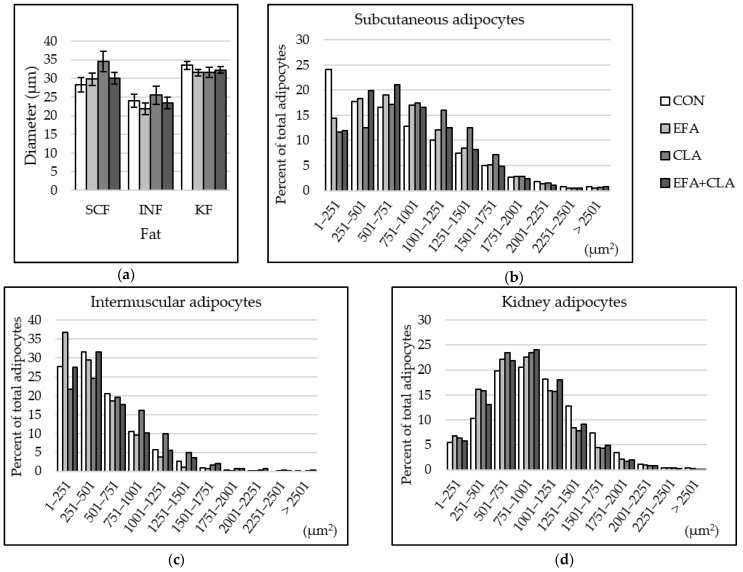
Fat cell size distribution in subcutaneous (SCF), intermuscular (INF) and kidney (KF) fat of calves of four maternal supplementation groups: Control group (CON, n = 8), essential fatty acids (EFA, n = 9), conjugated linoleic acids (CLA, n = 8), EFA+CLA (n = 11). (**a**) Fat cell diameter (µm). Data are shown as LSM ± SE_LSM_. (**b**–**d**) Percentage of adipocytes per size category (µm^2^) in (**b**) SCF, (**c**) INF, (**d**) KF.

The fat cell size was determined in different fat depots to elucidate whether the FA supple-mentation affected the development of adipocytes. The fat cell diameter was smaller in INF than in SCF and KF, but no group differences were observed (*p* > 0.05, Figure 6a). The histograms of the cell size distribution, however, revealed some changes. We determined the skewness factors that may indicate differences in cell size distribution among the groups. In SCF, the skewness coefficients of the four groups were 0.66 (CON), 0.14 (EFA), 0.15 (CLA), and 0.36 (EFA + CLA). Cells of supplemented animals, especially CLA, showed larger cells, compared to the control samples (Figure 6b). In INF, the skewness coefficients were 1.11 (CON), 1.37 (EFA), 0.55 (CLA), and 1.24 (EFA + CLA). More fat cells of smaller size were observed than in the other two tissues, and more small cells were measured in EFA supplemented animals than in the other groups (Figure 6c). This observation is supported by a negative correlation of fat cell diameter with tissue percentage of ALA (Spearman correlation coefficient: −0.42) and total *n*-3 PUFAs (Spearman correlation coefficient: −0.44, *p* < 0.05). A nearly normal size distribution was determined in kidney fat, without group differences (Figure 6d). The skewness coefficients were 0.39 (CON) and 0.56–0.67 (supplementation groups).

## 4. Discussion

A prerequisite for FA effects on cellular processes of muscle and adipose tissue development is the effective transfer of supplemented EFA and CLA from the cow to the offspring. It depends on the transport of FA via the placenta and colostrum to the fetus and the neonate, respectively [18]. Recent results indicate the desirable differences in the plasma FA composition of the respective calf treatment groups. Differences for ALA enrichment in plasma fat of EFA-treated calves were much greater on day 5 of life than immediately after birth, pointing at the importance of ALA transfer via milk feeding [34]. Thus, the tissue FA composition of the calves could have changed by maternal supplementation as intended, with consequences for the cellular development.

The data of the presented study confirmed that maternal supplementation with CLA and ALA elevated the levels of these fatty acids including de novo synthesized long-chain PUFAs in all investigated tissues of the calves. Furthermore, the supplementation elevated ALA metabolites. The reason for an increased enrichment of ALA metabolites in muscle and fat tissue is most likely due to a higher intake via placenta and milk, as the Δ−5 and Δ−6 desaturase indices were lower in EFA than non-EFA treated calves and DHA in plasma fat was already elevated in EFA-treated calves immediately after birth (Uken and Hammon, unpublished data). Even placental transfer of PUFA is low, an increased proportion of DHA but not ALA in plasma fat of newborn calves was already observed [35]. Thus, the observed changes in FA composition could have affected the cellular development in respective tissues. 

Generally, it is known that diet is the major factor influencing the fatty acid composition of ruminant tissues. Feeding *n*-3 PUFA-rich diets, such as grass, grass silage, and/or concentrates containing linseed, fish oil, or marine algae, results in beneficial enhancement of *n*-3 long-chain PUFAs and a reduction of SFA contents [36,37]. *N*-3 PUFA intervention caused reduced gene expression (mRNA- and protein level) of lipogenic enzymes (SREBP-1c, ACC, FAS, SCD-1) in beef muscle and resulted in lower concentrations of de novo synthesized MUFAs and SFAs [38]. However, *n*-3 PUFA intervention revealed tissue-specificities in fatty acid composition in beef cattle indicating that underlying mechanisms have not been completely understood until today.

Cells utilize FA in different ways, such as storage as triglycerides, incorporation into membranes, oxidation for energy generation, etc., but FA can also serve as ligands and signaling molecules for transcriptional events [39]. According to studies in cell culture models, intermediate products of FA metabolism are important for the survival, proliferation, differentiation, and fusion of myoblasts (e.g., [40,41]). For these processes, the lipid content of plasma membranes is important, regulating cell growth and metabolic activity of single cells. It is directly influenced by the lipid composition of the diet [42,43]. The balance between *n*-3 and *n*-6 PUFAs in the diet affects physical properties and lipid protein interactions in muscle cell membranes in particular [44].

Cows in the presented study were supplemented from the ninth week before the estimated parturition. At that time point, the primary and secondary muscle fibers are already developed in the fetus and mostly hypertrophy occurs [45,46]. At the time of birth, the muscle fiber number is fixed [47], while muscle metabolism and contractile properties are still subject to changes [29,48]. Fiber type conversion has been shown at the morphological level of mATPase staining, either from IIA to IIB fibers [29], or towards type I fibers [49,50]. Our data did not indicate that maternal FA supplementation caused changes in the total number and contraction type of muscle fibers. However, this may only be detectable over a longer period and not in calves shortly after birth as in the current study. Additionally, we attempted to detect possible fiber type transitions via detection of different myosin heavy chain isoforms in muscle tissue. Myosin heavy chain proteins work as markers for muscle fiber types; fast and intermediate fibers mostly express isoform MYH2, and slow fibers mostly express isoform MYH7. We found a strong correlation of the muscle fiber type in the myofibrillar ATPase staining with the area percentage of MYH2 and MYH7. Additionally, we found fibers expressing both isoforms. Muscle fibers expressing several MYH isoforms have been described before [51,52,53]. It is assumed that they are in transition between fiber types [53]. Our results indicated such changes in fiber types, but no influence of the maternal FA supplementation.

As a result of CLA supplementation in pigs, Men et al. [54] reported an increase in MYH7 mRNA expression. Slow muscle fibers store and metabolize more lipids than fast fibers [55,56]. There was no indication for more slow muscle fibers or increased incorporation of excess FA in muscle fibers in our study. Muscle fibers with visible lipid droplets were observed (data not shown), but the droplet size and number was too small to be measured.

Additionally, capillary density and fiber-capillary ratio were not influenced by supplementation, despite the evidence that prostaglandin metabolites of *n*-6 PUFAs promote angiogenic processes, while *n*-3 PUFA derived prostaglandins inhibit them [57,58]. The duration of supplementation or the FA dose might not have been sufficient to cause such changes.

Initial events of adipose tissue development start slightly later than primary myogenesis, like the formation of a vascular network to provide the basis for supply of cells with nutrients and signaling molecules [59,60,61]. Commitment of preadipocytes overlaps with the period of secondary myogenesis, which initiates around the end of the first trimester in ruminant animals [16]. Fatty acids are ligands for important transcription factors like the PPAR family members [62,63]. As such, they are involved in basic cellular processes during tissue development. Oxidized FA activate in particular PPARγ, a key regulator of adipogenesis, with greater potency than native FA [64]. Several studies in primary adipocyte culture and adipose tissue explants have shown that FA can modulate adipogenic and lipogenic processes (e.g., [65,66,67]).

Intramuscular adipocyte area was low, as expected in neonatal dairy calves (reviewed by Robelin [68]), and showed a high variance between samples. In pigs, fat deposition in the tissue (“marbling”) was increased by dietary CLA [69,70] and by *n*-3 PUFAs [71]. According to Cooke et al. [72], rumen-protected PUFAs increased marbling in cattle, while in other studies it did not have any effect [73,74]. Zhang et al. [75] reported higher intramuscular fat contents in cattle upon supplementation with CLA. These studies were conducted on animals older than the calves in this study. Neonatal calves might be too young to see any effects, because their intramuscular adipocytes are mostly just developing [76]. However, the shift to larger fat cells in SCF and INF of maternally-supplemented calves may point to the same direction. In these animals, excess PUFAs might be stored in the adipocytes. Further studies will show whether maternal supplementation has changed the recruitment and availability of preadipocytes and adipogenic factors.

## 5. Conclusions

The study showed that maternal supplementation with CLA and EFA changed the FA composition of skeletal muscle and adipose tissues of calves according to the supplementation. The elevation of these FA and some de novo synthesized longer-chain PUFAs was either not high enough to cause morphological or physiological changes in skeletal muscle or the animals were too young to see more than only minor changes in adipocyte size profiles. Consequences for recruitment of precursor cells or immune cells and therefore for later tissue development and health should be further investigated.

## Figures and Tables

**Table 1:** Fatty acid concentration (mg/100 g tissue) in longissimus muscle of calves of four maternal supplementation groups: Control group (CON, n = 8), essential fatty acids (EFA, n = 9), conjugated linoleic acids (CLA, n = 8), EFA + CLA (n = 11). Data are given as LSM ± SE_LSM_. Results for all measured fatty acids are listed in Supplementary Table S1.

Fatty Acid	Supplementation Group								Effect (*p*-Value)		
	CON		EFA		CLA		EFA + CLA		EFA	CLA	EFA × CLA
**C18:2 *c*-9, *t*-11 (CLA)**	**1.10**	**±0.10 ^b^**	**0.85**	±0.09 ^b^	1.85	±0.14 ^a^	1.65	±0.08 ^a^	0.036	<0.001	0.815
C18:3 *n*-3 (ALA)	2.03	±1.04 ^c^	11.22	±0.91 ^b^	1.99	±1.47 ^c^	15.19	±0.86 ^a^	<0.001	0.073	0.066
C20:5 *n*-3 (EPA)	1.89	±0.78 ^b^	8.73	±0.69 ^a^	1.67	±1.08 ^b^	10.24	±0.66 ^a^	<0.001	0.409	0.265
C22:5 *n*-3 (DPA)	8.72	±1.07 ^b^	16.55	±0.98 ^a^	8.64	±1.54 ^b^	19.32	±0.91 ^a^	<0.001	0.250	0.232
C22:6 *n*-3 (DHA)	5.79	±0.95 ^b^	9.44	±0.86 ^a^	4.96	±1.27 ^b^	9.65	±0.82 ^a^	<0.001	0.718	0.552
Sum *n*-3 PUFA ^1^	18.55	±3.14 ^b^	46.15	±2.78 ^a^	17.44	±4.45 ^b^	54.97	±2.62 ^a^	<0.001	0.233	0.128
C18:2 *n*-6 (LA)	63.30	±6.43	63.51	±5.70	75.05	±9.11	78.58	±5.39	0.781	0.048	0.798
C20:4 *n*-6 (ARA)	67.29	±4.20 ^ab^	57.23	±3.80 ^b^	75.42	±5.59 ^a^	62.91	±3.64 ^ab^	0.008	0.077	0.749
C22:4 *n*-6 (ADA)	19.58	±1.33 ^a^	13.53	±1.20 ^b^	22.27	±1.79 ^a^	15.07	±1.14 ^b^	<0.001	0.093	0.642
Sum *n*-6 PUFA ^2^	181.82	±12.68	163.08	±11.31	204.08	±17.71	188.74	±10.71	0.196	0.066	0.893
Sum PUFA ^3^	202.77	±14.83	211.39	±13.21	225.11	±20.81	246.61	±12.49	0.329	0.062	0.666
C18:1 *c*-9 (OA)	270.83	±12.51 ^ab^	240.65	±11.59 ^b^	297.36	±15.71 ^a^	247.38	±11.24 ^b^	0.001	0.099	0.321
C18:1 *t*-11 (VA)	0.45	±0.15	0.32	±0.13	0.48	±0.21	0.39	±0.13	0.474	0.749	0.892
Sum MUFA ^4^	349.86	±15.98 ^ab^	313.24	±14.79 ^b^	383.29	±20.10 ^a^	320.03	±14.34 ^b^	0.001	0.120	0.299
C16:0 (PA)	147.14	±9.41	128.36	±8.81	148.85	±11.51	134.83	±8.59	0.029	0.552	0.731
C18:0 (SA)	93.25	±5.49	87.85	±4.89	104.17	±7.64	104.22	±4.64	0.633	0.017	0.616
Sum SFA ^5^	257.48	±14.36	230.10	±13.26	269.91	±18.16	255.19	±12.85	0.086	0.111	0.585
Total fat content (%)	0.81	±0.04	0.76	±0.04	0.87	±0.05	0.82	±0.04	0.143	0.066	0.997

^1^ Sum *n*-3 PUFA: C18:3 *n*-3 + C20:3 *n*-3 + C20:5 *n*-3 + C22:5 *n*-3 + C22:6 *n*-3. ^2^ Sum *n*-6 PUFA: C18:2 *n*-6 + C18:3 *n*-6 + C20:2 *n*-6 + C20:3 *n*-6 + C20:4 *n*-6 + C22:4 *n*-6 + C22:5 *n*-6. ^3^ Sum PUFA: C18:2 *c*-9, *t*-11 + C18:2 *t*-9, *t*-12 + Sum *n*-3 PUFA + Sum *n*-6 PUFA. ^4^ Sum MUFA: C16:1 *c*-9 + C17:1 *c*-9 + C18:1 *c*-9 + C18:1 *c*-11 + C18:1 *t*-9 + C18:1 *t*-11 + C20:1 *c*-11 + C22:1 *c*-13. ^5^ Sum SFA: C10:0 + C12:0 + C14:0 + C15:0 + C16:0 + C17:0 + C18:0 + C20:0 + C21:0. ^a,b,c^ Different superscript letters indicate significant differences at *p* < 0.05.

**Table 2:** Fatty acid composition (% of total fatty acids) of subcutaneous fat of calves of four maternal supplementation groups: Control group (CON, n = 8), essential fatty acids (EFA, n = 9), conjugated linoleic acids (CLA, n = 8), EFA + CLA (n = 10). Data are given as LSM ± SE_LSM_. Results for all measured fatty acids are listed in Supplementary Table S3.

Fatty Acid	Supplementation Group								Effect (*p*-Value)		
	CON		EFA		CLA		EFA + CLA		EFA	CLA	EFA × CLA
C18:2 *c*-9, *t*-11 (CLA)	0.12	±0.02 ^b^	0.09	±0.01 ^b^	0.20	±0.02 ^a^	0.17	±0.01 ^a^	0.102	<0.001	0.958
C18:3 *n*-3 (ALA)	0.10	±0.05 ^b^	0.35	±0.04 ^a^	0.06	±0.07 ^b^	0.47	±0.04 ^a^	<0.001	0.484	0.167
C20:5 *n*-3 (EPA)	0.01	±0.01 ^b^	0.03	±0.00 ^a^	NA		0.03	±0.00 ^a^	<0.001	0.898	0.716
C22:5 *n*-3 (DPA)	0.08	±0.01 ^b^	0.14	±0.01 ^a^	NA		0.13	±0.01 ^ab^	<0.001	0.339	0.642
C22:6 *n*-3 (DHA)	0.03	±0.01	0.04	±0.00	NA		0.02	±0.00	0.067	0.049	0.686
Sum *n*-3 PUFA ^1^	0.22	±0.07 ^b^	0.57	±0.06 ^a^	0.10	±0.09 ^b^	0.68	±0.06 ^a^	<0.001	0.986	0.121
C18:2 *n*-6 (LA)	1.64	±0.15	1.78	±0.14	1.68	±0.22	1.78	±0.13	0.491	0.910	0.915
C20:4 *n*-6 (ARA)	0.30	±0.03	0.25	±0.03	0.24	±0.04	0.21	±0.03	0.218	0.164	0.795
C22:4 *n*-6 (ADA)	0.11	±0.01 ^a^	0.08	±0.01 ^ab^	0.09	±0.02 ^ab^	0.07	±0.01 ^b^	0.031	0.240	0.659
Sum *n*-6 PUFA ^2^	2.32	±0.19	2.37	±0.17	2.33	±0.27	2.30	±0.16	0.971	0.898	0.849
Sum PUFA ^3^	2.71	±0.25	3.07	±0.23	2.79	±0.36	3.19	±0.22	0.169	0.705	0.938
C18:1 *c*-9 (OA)	35.71	±0.90	33.82	±0.82	34.88	±1.29	33.97	±0.78	0.161	0.731	0.622
C18:1 *t*-11 (VA)	0.17	±0.03	0.14	±0.03	0.21	±0.04	0.18	±0.03	0.425	0.184	0.973
Sum MUFA ^4^	42.57	±0.93	40.72	±0.85	41.72	±1.34	40.27	±0.81	0.113	0.523	0.844
C16:0 (PA)	36.94	±0.80	38.01	±0.73	37.05	±1.14	37.35	±0.69	0.434	0.747	0.663
C18:0 (SA)	12.23	±0.34 ^b^	13.08	±0.30 ^ab^	12.77	±0.44 ^ab^	13.56	±0.29 ^a^	0.013	0.096	0.901
Sum SFA ^5^	54.72	±0.99	56.21	±0.91	55.49	±1.43	56.53	±0.86	0.248	0.614	0.839
Total fat content (g/100 g tissue)	16.09	±3.99	16.21	±3.52	16.31	±5.70	19.95	±3.40	0.659	0.633	0.666

^1^ Sum *n*-3 PUFA: C18:3 *n*-3 + C20:3 *n*-3 + C20:5 *n*-3 + C22:5 *n*-3 + C22:6 *n*-3. ^2^ Sum *n*-6 PUFA: C18:2 *n*-6 + C18:3 *n*-6 + C20:2 *n*-6 + C20:3 *n*-6 + C20:4 *n*-6 + C22:4 *n*-6. ^3^ Sum PUFA: C18:2 *c*-9, *t*-11 + C18:2 *t*-9, *t*-12 + C20:3 *n*-9 + Sum *n*-3 PUFA + Sum *n*-6 PUFA. ^4^ Sum MUFA: C14:1 *c*-9 + C16:1 *c*-9 + C17:1 *c*-9 + C18:1 *c*-9 + C18:1 *c*-11 + C18:1 *t*-9 + C18:1 *t*-11 + C20:1 *c*-11. ^5^ Sum SFA: C10:0 + C12:0 +C13:0 + C14:0 + C15:0 + C16:0 + C17:0 + C18:0 + C20:0 + C21:0 + C22:0 + C23:0 + C24:0. NA: LSM not calculated by SAS software due to small sample size. ^a,b^ Different superscript letters indicate significant differences at *p* < 0.05.

**Table 3:** Fatty acid concentration (mg/100 g tissue) in kidney fat of calves of four maternal supplementation groups: Control group (CON, n = 8), essential fatty acids (EFA, n = 9), conjugated linoleic acids (CLA, n = 8), EFA + CLA (n = 11). Data are given as LSM ± SE_LSM_. Results for all measured fatty acids are listed in Supplementary Table S5.

Fatty Acid	Supplementation Group								Effect (*p*-Value)		
	CON		EFA		CLA		EFA + CLA		EFA	CLA	EFA × CLA
C18:2 *c*-9, *t*-11 (CLA)	49.92	±6.30 ^ab^	35.58	±5.78 ^b^	68.13	±9.07 ^a^	62.58	±5.32 ^a^	0.154	0.002	0.527
C18:3 *n*-3 (LA)	37.86	±21.49 ^c^	120.27	±19.72 ^ab^	24.25	±30.95 ^bc^	179.10	±18.15 ^a^	<0.001	0.334	0.133
C18:4 *n*-3 (SDA)	2.48	±0.27 ^a^	1.51	±0.25 ^ab^	1.58	±0.39 ^ab^	1.31	±0.23 ^b^	0.044	0.070	0.244
C20:5 *n*-3 (EPA)	3.47	±0.82 ^b^	9.08	±0.73 ^a^	2.21	±1.17 ^b^	10.68	±0.69 ^a^	<0.001	0.842	0.100
C22:5 *n*-3 (DPA)	24.36	±2.50 ^b^	38.88	±2.30 ^a^	15.36	±3.61 ^b^	45.30	±2.11 ^a^	<0.001	0.635	0.009
C22:6 *n*-3 (DHA)	13.54	±2.18	19.78	±1.92	12.59	±3.20	20.00	±1.81	0.008	0.874	0.798
Sum *n*-3 PUFA ^1^	84.62	±23.39 ^b^	200.15	±21.46 ^a^	58.14	±33.69 ^b^	270.20	±19.75 ^a^	<0.001	0.391	0.069
C18:2 *n*-6 (LA)	571.82	±56.81	594.53	±52.12	549.65	±81.81	663.59	±47.97	0.274	0.702	0.467
C20:4 *n*-6 (ARA)	113.92	±8.34	99.50	±7.65	118.28	±12.01	107.55	±7.04	0.173	0.492	0.841
C22:4 *n*-6 (ADA)	45.67	±4.96	38.90	±4.55	49.34	±7.14	44.30	±4.19	0.279	0.400	0.874
Sum *n*-6 PUFA ^2^	884.34	±71.73	890.02	±65.81	862.91	±103.30	978.00	±60.57	0.441	0.668	0.490
Sum PUFA ^3^	1035	±88	1142	±81	1006	±127	1326	±75	0.033	0.420	0.277
C18:1 *c*-9 (OA)	22614	±957	23,365	±839	22,018	±1374	23,662	±795	0.246	0.880	0.648
C18:1 *t*-11 (VA)	58.18	±8.34	44.99	±7.66	50.03	±12.02	57.02	±7.05	0.733	0.829	0.277
Sum MUFA ^4^	29,499	±1161	30750	±1028	28,989	±1621	29,717	±977	0.408	0.504	0.820
C16:0 (PA)	20,678	±917	19976	±829	19,867	±1211	20,328	±796	0.886	0.778	0.479
C18:0 (SA)	6301	±485	6071	±442	6591	±628	6821	±426	1.000	0.214	0.580
Sum SFA ^5^	29,546	±1294	28193	±1194	28,950	±1632	29,329	±1158	0.649	0.793	0.406
Total fat content (%)	60.32	±1.87	60.09	±1.72	59.06	±2.36	60.46	±1.67	0.707	0.764	0.587

^1^ Sum *n*-3 PUFA: C18:3 *n*-3+ C18:4 *n*-3 + C20:3 *n*-3 + C20:5 *n*-3 + C22:5 *n*-3 + C22:6 *n*-3. ^2^ Sum *n*-6 PUFA: C18:2 *n*-6 + C18:3 *n*-6 + C20:2 *n*-6 + C20:3 *n*-6 + C20:4 *n*-6 + C22:2 *n*-6 + C22:4 *n*-6. ^3^ Sum PUFA: C18:2 *c*-9, *t*-11 + C18:2 *t*-9, *t*-12 + C20:3 *n*-9 + Sum *n*-3 PUFA + Sum *n*-6 PUFA. ^4^ Sum MUFA: C14:1 *c*-9 + C16:1 *c*-9 + C17:1 *c*-9 + C18:1 *c*-9 + C18:1 *c*-11 + C18:1 *t*-9 + C18:1 *t*-11 + C20:1 *c*-11 + C22:1 *c*-13 + C24:1 *c*-15. ^5^ Sum SFA: C10:0 + C11:0 + C12:0 + C13:0 + C14:0 + C15:0 + C16:0 + C17:0 + C18:0 + C20:0 + C21:0 + C22:0 + C23:0 + C24:0. ^a,b,c^ Different superscript letters indicate significant differences at *p* < 0.05.

## Supplementary Materials

The following are available online at https://www.mdpi.com/2076-2615/10/9/1598/s1, **Table S1.** Fatty acid concentration (mg/100 g tissue) in longissimus muscle. **Table S2.** Fatty acid concentration (mg/100 g tissue) in semitendinosus muscle. **Table S3.** Fatty acid composition (% of total fatty acids) in subcutaneous fat. **Table S4.** Fatty acid composition (% of total fatty acids) in intermuscular fat (at longissimus muscle). **Table S5.** Fatty acid concentration (mg/100 g tissue) in kidney fat. **Figure S1.** Number of nuclei per muscle fiber of each fiber type in longissimus and semitendinosus muscle. **Figure S2.** Representative western blot pictures of myosin heavy chain isoforms 1, 2, and 7 and total protein in skeletal muscle.
