# Supplementary material for: Consequences of Maternal Essential Fatty Acid and Conjugated Linoleic Acid Supplementation on the Development of Calf Muscle and Adipose Tissue
Source: Animals (Basel). 2020 Sep 8;10(9):1598. doi: 10.3390/ani10091598 (PMC7552164; doi:10.3390/ani10091598)
Supplement: Supplementary file 1 [file animals-10-01598-s001.pdf]

animals

MDPI

Supplementary Materials

Table 1. Fatty acid concentration (mg/100 g tissue) in longissimus muscle of calves of four maternal supplementation groups: Control group (CON, n = 8), essential fatty acids (EFA, n = 9), conjugated linoleic acids (CLA, n = 8), EFA + CLA (n = 11). Data are given as LSM ± SE_{LSM}.

Fatty Acid	Supplementation Group								Effect (*p*-Value)		
	CON		EFA		CLA		EFA + CLA		EFA	CLA	EFA * CLA
C18:2 *c*-9, *t*-11	1.10	± 0.10 [b]	0.85	± 0.09 [b]	1.85	± 0.14 [a]	1.65	± 0.08 [a]	0.036	<.001	0.815
C18:3 *n*-3	2.03	± 1.04 [c]	11.22	± 0.91 [b]	1.99	± 1.47 [c]	15.19	± 0.86 [a]	<.001	0.073	0.066
C20:5 *n*-3	1.89	± 0.78 [b]	8.73	± 0.69 [a]	1.67	± 1.08 [b]	10.24	± 0.66 [a]	<.001	0.409	0.265
C22:5 *n*-3	8.72	± 1.07 [b]	16.55	± 0.98 [a]	8.64	± 1.54 [b]	19.32	± 0.91 [a]	<.001	0.250	0.232
C22:6 *n*-3	5.79	± 0.95 [b]	9.44	± 0.86 [a]	4.96	± 1.27 [b]	9.65	± 0.82 [a]	<.001	0.718	0.552
Sum *n*-3 PUFA [1]	18.55	± 3.14 [b]	46.15	± 2.78 [a]	17.44	± 4.45 [b]	54.97	± 2.62 [a]	<.001	0.233	0.128
C18:2 *n*-6	63.30	± 6.43	63.51	± 5.70	75.05	± 9.11	78.58	± 5.39	0.781	0.048	0.798
C18:3 *n*-6	1.21	± 0.15	1.18	± 0.14	1.12	± 0.21	1.28	± 0.13	0.680	0.969	0.514
C20:3 *n*-6	27.80	± 2.22	25.87	± 2.04	26.34	± 3.20	28.50	± 1.88	0.962	0.806	0.406
C20:4 *n*-6	67.29	± 4.20 [ab]	57.23	± 3.80 [b]	75.42	± 5.59 [a]	62.91	± 3.64 [ab]	0.008	0.077	0.749
C22:4 *n*-6	19.58	± 1.33 [a]	13.53	± 1.20 [b]	22.27	± 1.79 [a]	15.07	± 1.14 [b]	<.001	0.093	0.642
C22:5 *n*-6	1.55	± 0.25 [a]	0.65	± 0.23 [b]	1.87	± 0.28 [a]	0.71	± 0.26 [b]	<.001	0.270	0.424
Sum *n*-6 PUFA [2]	181.82	± 12.68	163.08	± 11.31	204.08	± 17.71	188.74	± 10.71	0.196	0.066	0.893
C18:2 *t*-9, *t*-12	1.28	± 0.15	1.37	± 0.14	1.51	± 0.18	1.14	± 0.14	0.178	0.988	0.031
C20:3 *n*-3	NA		0.26	± 0.07 [b]	NA		0.58	± 0.05 [a]	0.964	0.002	NA
C20:2 *n*-6	1.20	± 0.17 [ab]	1.12	± 0.16 [b]	1.54	± 0.25 [ab]	1.75	± 0.15 [a]	0.724	0.015	0.445
Sum PUFA [3]	202.77	± 14.83	211.39	± 13.21	225.11	± 20.81	246.61	± 12.49	0.329	0.062	0.666
C16:1 *c*-9	27.77	± 1.74 [ab]	24.59	± 1.57 [ab]	31.26	± 2.34 [a]	22.97	± 1.50 [b]	0.002	0.559	0.120
C17:1 *c*-9	7.88	± 0.82	7.22	± 0.74	8.71	± 1.09	7.36	± 0.70	0.199	0.512	0.647
C18:1 *c*-9	270.83	± 12.51 [ab]	240.65	± 11.59 [b]	297.36	± 15.71 [a]	247.38	± 11.24 [b]	0.001	0.099	0.321
C18:1 *c*-11	39.78	± 2.38	37.77	± 2.14	42.42	± 3.22	39.32	± 2.04	0.272	0.349	0.808
C18:1 *t*-9	1.08	± 0.09 [ab]	0.90	± 0.08 [b]	1.42	± 0.12[a]	0.99	± 0.07 [b]	0.001	0.014	0.127
C18:1 *t*-11	0.45	± 0.15	0.32	± 0.13	0.48	± 0.21	0.39	± 0.13	0.474	0.749	0.892
C20:1 *c*-11	1.89	± 0.12	1.61	± 0.11	1.90	± 0.18	1.80	± 0.10	0.174	0.457	0.508

C22:1 *c*-13	0.14	± 0.02	NA		0.19	± 0.03	0.16	± 0.04	0.541	0.209	NA
Sum MUFA [4]	349.86	± 15.98 [ab]	313.24	± 14.79 [b]	383.29	± 20.10 [a]	320.03	± 14.34 [b]	0.001	0.120	0.299
C10:0	0.49	± 0.07	0.55	± 0.07	0.61	± 0.08	0.50	± 0.07	0.465	0.328	0.013
C12:0	0.44	± 0.08	0.50	± 0.07	0.47	± 0.11	0.55	± 0.07	0.370	0.655	0.876
C14:0	7.54	± 0.81 [a]	4.54	± 0.72 [b]	6.80	± 1.12 [ab]	6.26	± 0.68 [ab]	0.038	0.534	0.128
C15:0	1.28	± 0.09	1.06	± 0.08	1.34	± 0.12	1.26	± 0.07	0.085	0.120	0.401
C16:0	147.14	± 9.41	128.36	± 8.81	148.85	± 11.51	134.83	± 8.59	0.029	0.552	0.731
C17:0	5.64	± 0.32	5.22	± 0.30	5.89	± 0.47	6.11	± 0.27	0.781	0.113	0.381
C18:0	93.25	± 5.49	87.85	± 4.89	104.17	± 7.64	104.22	± 4.64	0.633	0.017	0.616
C20:0	0.83	± 0.08	0.78	± 0.07	1.09	± 0.11	0.84	± 0.07	0.047	0.026	0.172
C21:0 [5]	0.20	± 0.06	0.11	± 0.05	0.33	± 0.07	0.28	± 0.05	0.249	0.021	0.730
Sum SFA [6]	257.48	± 14.36	230.10	± 13.26	269.91	± 18.16	255.19	± 12.85	0.086	0.111	0.585
Total fat content (%)	0.81	± 0.04	0.76	± 0.04	0.87	± 0.05	0.82	± 0.04	0.143	0.066	0.997

[1] Sum *n*-3 PUFA: C18:3 *n*-3 + C20:3 *n*-3 + C20:5 *n*-3 + C22:5 *n*-3 + C22:6 *n*-3. [2] Sum *n*-6 PUFA: C18:2 *n*-6 + C18:3 *n*-6 + C20:2 *n*-6 + C20:3 *n*-6 + C20:4 *n*-6 + C22:4 *n*-6 + C22:5 *n*-6. [3] Sum PUFA: C18:2 *c*-9, *t*-11 + C18:2 *t*-9, *t*-12 + Sum *n*-3 PUFA + Sum *n*-6 PUFA. [4] Sum MUFA: C16:1 *c*-9 + C17:1 *c*-9 + C18:1 *c*-9 + C18:1 *c*-11 + C18:1 *t*-9 + C18:1 *t*-11 + C20:1 *c*-11 + C22:1 *c*-13. [5] C21:0: includes *t*-10, *c*-12 CLA. [6] Sum SFA: C10:0 + C12:0 + C14:0 + C15:0 + C16:0 + C17:0 + C18:0 + C20:0 + C21:0. [a,b,c] Different superscript letters indicate significant differences at $p < 0.05$.

Table 2. Fatty acid concentration (mg/100 g tissue) in semitendinosus muscle of calves of four maternal supplementation groups: Control group (CON, n = 8), essential fatty acids (EFA, n = 9), conjugated linoleic acids (CLA, n = 8), EFA + CLA (n = 11). Data are given as LSM ± SE_{LSM}.

Fatty Acid	Supplementation Group								Effect (*p*-Value)		
	CON		EFA		CLA		EFA + CLA		EFA	CLA	EFA * CLA
C18:2 *c*-9, *t*-11	0.91	± 0.12 [b]	0.72	± 0.11 [b]	1.67	± 0.17 [a]	1.66	± 0.10 [a]	0.447	<.001	0.494
C18:3 *n*-3	1.94	± 0.71 [c]	10.02	± 0.63 [b]	2.04	± 0.98 [c]	13.00	± 0.60 [a]	<.001	0.032	0.043
C18:4 *n*-3	0.33	± 0.03	0.32	± 0.03	0.35	± 0.04	0.32	± 0.03	0.486	0.802	0.846
C20:5 *n*-3	1.60	± 0.35 [b]	5.73	± 0.31 [a]	1.34	± 0.47 [b]	6.49	± 0.29 [a]	<.001	0.458	0.133
C22:5 *n*-3	8.55	± 0.68 [b]	15.72	± 0.62 [a]	7.80	± 0.97 [b]	17.60	± 0.57 [a]	<.001	0.441	0.085
C22:6 *n*-3	4.53	± 0.57 [b]	7.03	± 0.50 [a]	3.88	± 0.81 [b]	7.23	± 0.47 [a]	<.001	0.698	0.467
Sum *n*-3 PUFA [1]	17.29	± 1.46 [c]	39.34	± 1.34 [b]	15.62	± 2.10 [c]	45.44	± 1.23 [a]	<.001	0.165	0.021
C18:2 *n*-6	55.58	± 4.78	59.89	± 4.20	63.15	± 6.89	71.24	± 3.97	0.232	0.065	0.701
C18:3 *n*-6	1.31	± 0.10	1.27	± 0.09	1.17	± 0.15	1.28	± 0.09	0.725	0.537	0.510
C20:3 *n*-6	29.66	± 2.13	29.59	± 1.95	26.54	± 3.07	30.38	± 1.80	0.418	0.613	0.406
C20:4 *n*-6	55.19	± 2.03	48.13	± 1.81	56.52	± 2.88	51.44	± 1.71	0.008	0.268	0.632
C22:4 *n*-6	15.20	± 0.76 [a]	11.23	± 0.67 [b]	15.73	± 1.04 [a]	11.99	± 0.64 [b]	<.001	0.380	0.876
C22:5 *n*-6	2.81	± 0.19	2.37	± 0.17	2.86	± 0.26	2.34	± 0.17	0.016	0.976	0.816
Sum *n*-6 PUFA [2]	161.34	± 7.51	153.88	± 6.89	167.99	± 10.82	171.39	± 6.34	0.804	0.144	0.512
C18:2 *t*-9, *t*-12	1.65	± 0.17	1.56	± 0.16	1.96	± 0.24	1.57	± 0.14	0.197	0.403	0.417
C20:3 *n*-3	NA		0.53	± 0.05 [b]	NA		0.85	± 0.05 [a]	<.001	<.001	NA
C20:2 *n*-6	1.65	± 0.20 [ab]	1.57	± 0.18 [b]	1.82	± 0.27 [ab]	2.19	± 0.17 [a]	0.478	0.044	0.244
C20:3 *n*-9	3.44	± 0.34	3.46	± 0.31	3.99	± 0.48	3.15	± 0.28	0.270	0.749	0.247
Sum PUFA [3]	184.63	± 7.95 [b]	198.97	± 7.29 [ab]	191.22	± 11.45 [ab]	223.20	± 6.71 [a]	0.012	0.081	0.317
C16:1 *c*-9	23.65	± 1.61	23.41	± 1.48	26.69	± 2.32	21.23	± 1.36	0.113	0.805	0.149
C17:1 *c*-9	2.41	± 0.28	1.67	± 0.26	2.36	± 0.39	1.68	± 0.24	0.015	0.940	0.906
C18:1 *c*-9	228.54	± 11.02	215.81	± 10.43	241.22	± 13.10	216.98	± 10.22	0.022	0.349	0.439
C18:1 *c*-11	33.22	± 1.42	34.08	± 1.25	33.80	± 2.00	34.78	± 1.18	0.533	0.655	0.967
C18:1 *t*-9	1.14	± 0.11 [ab]	1.06	± 0.09 [b]	1.55	± 0.14 [a]	1.24	± 0.09 [ab]	0.074	0.006	0.259
C18:1 *t*-11	1.32	± 0.10 [b]	1.20	± 0.09 [b]	1.51	± 0.14 [ab]	1.67	± 0.08 [a]	0.864	0.003	0.195
C20:1 *c*-11	2.13	± 0.16	1.92	± 0.14	1.97	± 0.20	2.10	± 0.14	0.748	0.911	0.203
C22:1 *c*-13	0.43	± 0.03	0.40	± 0.02	0.42	± 0.04	0.41	± 0.02	0.414	0.892	0.680

Sum MUFA [4]	293.08	± 13.41	279.61	± 12.58	309.84	± 16.33	279.85	± 12.28	0.038	0.381	0.398
C10:0	0.81	± 0.07	0.82	± 0.07	0.82	± 0.10	0.74	± 0.06	0.567	0.624	0.476
C12:0	1.05	± 0.10 ^{b}	0.65	± 0.09 ^{a}	0.76	± 0.14 ^{ab}	0.92	± 0.09 ^{ab}	0.227	0.936	0.007
C14:0	7.67	± 1.06	5.47	± 0.97	6.57	± 1.36	7.94	± 0.94	0.655	0.445	0.054
C15:0	1.35	± 0.12	1.36	± 0.10	1.37	± 0.15	1.51	± 0.10	0.491	0.407	0.554
C16:0	129.97	± 9.09	118.68	± 8.50	122.80	± 11.16	136.86	± 8.28	0.842	0.414	0.070
C17:0	7.99	± 0.55	7.66	± 0.51	7.89	± 0.69	8.90	± 0.49	0.452	0.192	0.129
C18:0	84.00	± 4.81 ^{b}	86.67	± 4.54 ^{b}	92.63	± 5.79 ^{ab}	98.49	± 4.43 ^{a}	0.224	0.005	0.635
C20:0	1.15	± 0.06 ^{b}	1.25	± 0.06 ^{ab}	1.35	± 0.09 ^{ab}	1.37	± 0.05 ^{a}	0.361	0.016	0.546
C21:0 [5]	0.36	± 0.05 ^{b}	0.34	± 0.05 ^{b}	0.69	± 0.07 ^{a}	0.77	± 0.04 ^{a}	0.643	<.001	0.386
C22:0	1.05	± 0.10	0.93	± 0.09	1.23	± 0.13	1.06	± 0.08	0.126	0.091	0.806
C23:0	0.67	± 0.08	0.67	± 0.07	0.97	± 0.11	0.73	± 0.06	0.141	0.027	0.132
Sum SFA [6]	236.24	± 15.15 ^{ab}	224.48	± 14.23 ^{b}	237.33	± 18.39 ^{ab}	259.29	± 13.89 ^{a}	0.649	0.105	0.130
Total fat content (%)	0.71	± 0.03	0.70	± 0.03	0.74	± 0.04	0.76	± 0.03	0.785	0.091	0.487

[1] Sum *n*-3 PUFA: C18:3 *n*-3 + C18:4 *n*-3 + C20:3 *n*-3 + C20:5 *n*-3 + C22:5 *n*-3 + C22:6 *n*-3. [2] Sum *n*-6 PUFA: C18:2 *n*-6 + C18:3 *n*-6 + C20:2 *n*-6 + C20:3 *n*-6 + C20:4 *n*-6 + C22:4 *n*-6 + C22:5 *n*-6. [3] Sum PUFA: C18:2 *c*-9, *t*-11 + C18:2 *t*-9, *t*-12 + C20:3 *n*-9 + Sum *n*-3 PUFA + Sum *n*-6 PUFA. [4] Sum MUFA: C16:1 *c*-9 + C17:1 *c*-9 + C18:1 *c*-9 + C18:1 *c*-11 + C18:1 *t*-9 + C18:1 *t*-11 + C20:1 *c*-11 + C22:1 *c*-13. [5] C21:0: includes *t*-10, *c*-12 CLA. [6] Sum SFA: C10:0 + C12:0 + C14:0 + C15:0 + C16:0 + C17:0 + C18:0 + C20:0 + C21:0 + C22:0 + C23:0. NA: Not calculated by SAS software due to small sample size. ^{a,b,c} Different superscript letters indicate significant differences at $p < 0.05$.

Table 3. Fatty acid composition (% of total fatty acids) in subcutaneous fat of calves of four maternal supplementation groups: Control group (CON, n = 8), essential fatty acids (EFA, n = 9), conjugated linoleic acids (CLA, n = 8), EFA + CLA (n = 10). Data are given as LSM ± SE_{LSM}.

Fatty Acid	Supplementation Group								Effect (*p*-Value)		
	CON		EFA		CLA		EFA + CLA		EFA	CLA	EFA*CLA
C18:2 *c*-9, *t*-11	0.12	± 0.02 [b]	0.09	± 0.01 [b]	0.20	± 0.02 [a]	0.17	± 0.01 [a]	0.102	<.001	0.958
C18:3 *n*-3	0.10	± 0.05 [b]	0.35	± 0.04 [a]	0.06	± 0.07 [b]	0.47	± 0.04 [a]	<.001	0.484	0.167
C20:5 *n*-3	0.01	± 0.01 [b]	0.03	± 0.00 [a]	NA		0.03	± 0.00 [a]	<.001	0.898	0.716
C22:5 *n*-3	0.08	± 0.01 [b]	0.14	± 0.01 [a]	NA		0.13	± 0.01 [ab]	<.001	0.339	0.642
C22:6 *n*-3	0.03	± 0.01	0.04	± 0.00	NA		0.02	± 0.00	0.067	0.049	0.686
Sum *n*-3 PUFA [1]	0.22	± 0.07 [b]	0.57	± 0.06 [a]	0.10	± 0.09 [b]	0.68	± 0.06 [a]	<.001	0.986	0.121
C18:2 *n*-6	1.64	± 0.15	1.78	± 0.14	1.68	± 0.22	1.78	± 0.13	0.491	0.910	0.915
C18:3 *n*-6	0.04	± 0.00 [b]	0.04	± 0.00[b]	0.11	± 0.01 [a]	0.03	± 0.00 [b]	<.001	<.001	<.001
C20:3 *n*-6	0.17	± 0.01	0.17	± 0.01	0.13	± 0.02	0.15	± 0.01	0.706	0.081	0.579
C20:4 *n*-6	0.30	± 0.03	0.25	± 0.03	0.24	± 0.04	0.21	± 0.03	0.218	0.164	0.795
C22:4 *n*-6	0.11	± 0.01 [a]	0.08	± 0.01 [ab]	0.09	0.02 [ab]	0.07	± 0.01 [b]	0.031	0.240	0.659
Sum *n*-6 PUFA [2]	2.32	± 0.19	2.37	± 0.17	2.33	± 0.27	2.30	± 0.16	0.971	0.898	0.849
C18:2 *t*-9, *t*-12	0.01	± 0.00 [b]	0.02	± 0.00 [b]	0.11	± 0.01 [a]	0.01	± 0.00 [b]	<.001	<.001	<.001
C20:3 *n*-3	NA		0.02	± 0.00 [b]	NA		0.03	± 0.00 [a]	0.003	0.048	NA
C20:2 *n*-6	0.06	± 0.01	0.06	± 0.01	0.07	± 0.01	0.06	± 0.01	0.608	0.117	0.365
C20:3 n-9	0.04	± 0.01	0.04	± 0.01	0.04	± 0.01	0.03	± 0.01	0.242	0.265	0.659
Sum PUFA [3]	2.71	± 0.25	3.07	± 0.23	2.79	± 0.36	3.19	± 0.22	0.169	0.705	0.938
C14:1 *c*-9	0.54	± 0.04	0.45	± 0.04	0.41	± 0.06	0.45	± 0.04	0.590	0.166	0.188
C16:1 *c*-9	3.90	± 0.23	4.13	± 0.21	3.85	± 0.33	3.64	± 0.20	0.966	0.286	0.391
C17:1 *c*-9	0.05	± 0.03	0.03	± 0.02	0.05	± 0.03	0.02	± 0.02	0.380	0.895	0.792
C18:1 *c*-9	35.71	± 0.90	33.82	± 0.82	34.88	± 1.29	33.97	± 0.78	0.161	0.731	0.622
C18:1 *c*-11	1.92	± 0.11	1.87	± 0.10	2.06	± 0.15	1.73	± 0.10	0.092	0.965	0.181
C18:1 *t*-9	0.16	± 0.01	0.14	± 0.01	0.13	± 0.02	0.16	± 0.01	0.788	0.850	0.157
C18:1 *t*-11	0.17	± 0.03	0.14	± 0.03	0.21	± 0.04	0.18	± 0.03	0.425	0.184	0.973
C20:1 *c*-11	0.14	± 0.01	0.13	± 0.01	0.13	± 0.01	0.13	± 0.01	0.450	0.717	0.628
Sum MUFA [4]	42.57	± 0.93	40.72	± 0.85	41.72	± 1.34	40.27	± 0.81	0.113	0.523	0.844
C10:0	0.09	± 0.01	0.09	± 0.01	0.08	± 0.02	0.10	± 0.01	0.346	0.743	0.523
C12:0	0.34	± 0.05	0.28	± 0.04	0.30	± 0.07	0.32	± 0.04	0.634	0.947	0.407

C13:0	0.02	± 0.00	0.02	± 0.00	0.02	± 0.00	0.02	± 0.00	0.207	0.582	0.704
C14:0	3.93	± 0.34	3.75	± 0.30	4.02	± 0.49	4.06	± 0.29	0.847	0.563	0.748
C15:0	0.26	± 0.03	0.25	± 0.03	0.34	± 0.04	0.29	± 0.03	0.351	0.066	0.418
C16:0	36.94	± 0.80	38.01	± 0.73	37.05	± 1.14	37.35	± 0.69	0.434	0.747	0.663
C17:0	0.66	± 0.03	0.63	± 0.03	0.71	± 0.05	0.66	± 0.03	0.281	0.257	0.802
C18:0	12.23	± 0.34 [b]	13.08	± 0.30 [ab]	12.77	± 0.44 [ab]	13.56	± 0.29 [a]	0.013	0.096	0.901
C20:0	0.08	± 0.00	0.08	± 0.00	0.08	± 0.01	0.08	± 0.00	0.468	0.497	0.694
C21:0 [5]	0.01	± 0.01 [b]	0.01	± 0.01 [b]	0.05	± 0.01 [a]	0.05	± 0.00 [a]	0.947	<.001	0.838
C22:0	0.02	± 0.00	0.02	± 0.00	NA		0.01	± 0.00	0.842	0.202	0.717
C23:0	0.02	± 0.01	0.01	± 0.00	0.02	± 0.01	0.01	± 0.00	0.066	0.778	0.952
C24:0	0.03	± 0.01	0.03	± 0.01	NA		0.01	± 0.01	0.795	0.305	0.486
Sum SFA [6]	54.72	± 0.99	56.21	± 0.91	55.49	± 1.43	56.53	± 0.86	0.248	0.614	0.839
Total fat content (g/100g tissue)	16.09	± 3.99	16.21	± 3.52	16.31	± 5.70	19.95	± 3.40	0.659	0.633	0.666

[1] Sum *n*-3 PUFA: C18:3 *n*-3 + C20:3 *n*-3 + C20:5 *n*-3 + C22:5 *n*-3 + C22:6 *n*-3. [2] Sum *n*-6 PUFA: C18:2 *n*-6 + C18:3 *n*-6 + C20:2 *n*-6 + C20:3 *n*-6 + C20:4 *n*-6 + C22:4 *n*-6. [3] Sum PUFA: C18:2 *c*-9, *t*-11 + C18:2 *t*-9, *t*-12 + C20:3 *n*-9 + Sum *n*-3 PUFA + Sum *n*-6 PUFA. [4] Sum MUFA: C14:1 *c*-9 + C16:1 *c*-9 + C17:1 *c*-9 + C18:1 *c*-9 + C18:1 *c*-11 + C18:1 *t*-9 + C18:1 *t*-11 + C20:1 *c*-11. [5] C21:0: includes *t*-10, *c*-12 CLA. [6] Sum SFA: C10:0 + C12:0 +C13:0 + C14:0 + C15:0 + C16:0 + C17:0 + C18:0 + C20:0 + C21:0 + C22:0 + C23:0 + C24:0. NA: Not calculated by SAS software due to small sample size. [a,b] Different superscript letters indicate significant differences at $p < 0.05$.

Table 4. Fatty acid composition (% of total fatty acids) in intermuscular fat of calves of four maternal supplementation groups: Control group (CON, n = 7), essential fatty acids (EFA, n = 8), conjugated linoleic acids (CLA, n = 5), EFA + CLA (n = 7). Data are given as LSM ± SE_{LSM}.

Fatty Acid	Supplementation Group								Effect (*p*-Value)		
	CON		EFA		CLA		EFA + CLA		EFA	CLA	EFA * CLA
C18:2 *c*-9, *t*-11	0.11	± 0.02 [ab]	0.08	± 0.02 [b]	0.13	± 0.03 [ab]	0.16	± 0.02 [a]	0.897	0.023	0.191
C18:3 *n*-3	0.14	± 0.07 [bc]	0.40	± 0.06 [ab]	0.06	± 0.10 [c]	0.48	± 0.07 [a]	<.001	0.951	0.313
C18:4 *n*-3	0.02	± 0.01	0.03	± 0.01	NA		0.02	± 0.01	0.190	0.084	0.343
C20:5 *n*-3	0.02	± 0.02	0.07	± 0.02	0.00	± 0.02	0.06	± 0.02	0.005	0.293	0.643
C22:5 *n*-3	0.21	± 0.08	0.25	± 0.07	0.05	± 0.10	0.24	± 0.08	0.132	0.221	0.295
C22:6 *n*-3	0.05	± 0.02	0.06	± 0.02	0.01	± 0.03	0.05	± 0.02	0.247	0.245	0.372
Sum *n*-3 PUFA [1]	0.48	± 0.16 [ab]	0.81	± 0.14 [ab]	0.16	± 0.23 [b]	0.88	± 0.15 [c]	0.006	0.460	0.243
C18:2 *n*-6	2.08	± 0.32	1.86	± 0.28	1.57	± 0.43	2.13	± 0.30	0.583	0.703	0.205
C18:3 *n*-6	0.04	± 0.01	0.03	± 0.01	0.02	± 0.01	0.03	± 0.01	0.981	0.539	0.142
C20:3 *n*-6	0.47	± 0.08	0.30	± 0.07	0.18	± 0.11	0.30	± 0.08	0.753	0.072	0.082
C20:4 *n*-6	1.55	± 0.36	0.77	± 0.32	0.43	± 0.48	0.77	± 0.34	0.529	0.114	0.111
C22:4 *n*-6	0.51	± 0.11	0.20	± 0.09	0.16	± 0.16	0.25	± 0.10	0.343	0.180	0.087
Sum *n*-6 PUFA [2]	4.74	± 0.81	3.26	± 0.72	2.46	± 1.09	3.59	± 0.78	0.825	0.214	0.098
C18:2 *t*-9, *t*-12	0.03	± 0.01	0.03	± 0.01	0.02	± 0.02	0.03	± 0.01	0.673	0.873	0.628
C20:3 *n*-3	0.01	± 0.01 [b]	0.03	± 0.00 [ab]	NA		0.03	± 0.01 [a]	0.023	0.486	0.870
C20:2 *n*-6	0.08	± 0.01	0.07	± 0.01	0.06	± 0.02	0.08	± 0.01	0.548	0.887	0.186
C22:2 *n*-6	NA		NA		0.01	± 0.01	NA		0.453	0.116	NA
C20:3 *n*-9	0.09	± 0.02	0.05	± 0.01	0.04	± 0.02	0.05	± 0.02	0.464	0.168	0.133
Sum PUFA [3]	5.20	± 0.95	4.06	± 0.84	2.61	± 1.28	4.46	± 0.91	0.702	0.233	0.107
C14:1 *c*-9	0.46	± 0.08	0.49	± 0.07	0.43	± 0.11	0.33	± 0.08	0.657	0.237	0.402
C16:1 *c*-9	3.68	± 0.49	4.15	± 0.43	4.07	± 0.70	2.79	± 0.47	0.448	0.345	0.095
C17:1 *c*-9	0.33	± 0.14	0.17	± 0.12	0.13	± 0.20	0.18	± 0.13	0.695	0.521	0.466
C18:1 *c*-9	34.46	± 0.94	33.35	± 0.77	34.45	± 1.34	32.84	± 0.87	0.182	0.780	0.782
C18:1 *c*-11	2.03	± 0.13	1.86	± 0.11	1.91	± 0.17	1.84	± 0.12	0.365	0.582	0.694
C18:1 *t*-9	0.16	± 0.02	0.13	± 0.02	0.12	± 0.03	0.16	± 0.02	0.696	0.686	0.166
C18:1 *t*-11	0.22	± 0.03	0.16	± 0.03	0.12	± 0.04	0.18	± 0.03	0.993	0.298	0.137
C20:1 *c*-11	0.24	± 0.03	0.18	± 0.03	0.16	± 0.05	0.19	± 0.03	0.634	0.356	0.184
C22:1 *c*-13	0.05	± 0.01	0.02	± 0.01	0.01	± 0.02	0.03	± 0.01	0.633	0.206	0.117

Sum MUFA [4]	41.59	± 1.01	40.54	± 0.84	41.38	± 1.42	38.53	± 0.94	0.076	0.278	0.359
C10:0	0.12	± 0.02	0.09	± 0.02	0.08	± 0.03	0.10	± 0.02	0.801	0.426	0.229
C12:0	0.35	± 0.06	0.31	± 0.06	0.22	± 0.09	0.32	± 0.06	0.624	0.353	0.312
C13:0	0.03	± 0.01	0.02	± 0.00	0.02	± 0.01	0.02	± 0.00	0.605	0.311	0.168
C14:0	3.69	± 0.45	3.77	± 0.39	3.79	± 0.59	3.86	± 0.43	0.855	0.823	0.992
C15:0	0.27	± 0.04	0.26	± 0.03	0.25	± 0.05	0.29	± 0.03	0.721	0.953	0.391
C16:0	34.16	± 1.34	35.76	± 1.19	36.52	± 1.78	35.66	± 1.29	0.769	0.369	0.321
C17:0	1.25	± 0.12	1.22	± 0.10	1.31	± 0.16	1.47	± 0.11	0.579	0.182	0.394
C18:0	12.78	± 0.60 [b]	13.54	± 0.54 [ab]	13.46	± 0.76 [ab]	14.70	± 0.58 [a]	0.068	0.083	0.633
C20:0	0.19	± 0.03	0.12	± 0.03	0.09	± 0.05	0.14	± 0.03	0.826	0.268	0.111
C21:0 [5]	0.09	± 0.03	0.02	± 0.02	0.05	± 0.04	0.07	± 0.03	0.318	0.976	0.116
C22:0	0.10	± 0.03	0.04	± 0.02	0.03	± 0.04	0.05	± 0.03	0.385	0.245	0.129
C23:0	0.03	± 0.01	0.03	± 0.01	0.03	± 0.01	0.02	± 0.01	0.874	0.285	0.349
C24:0	0.02	± 0.01	0.01	± 0.00	0.01	± 0.01	0.02	± 0.00	0.878	0.423	0.287
Sum SFA [6]	53.12	± 1.26	55.20	± 1.08	55.93	± 1.71	56.78	± 1.20	0.249	0.082	0.595
Total fat content (%)	10.74	± 3.94	9.78	± 3.89	13.36	± 5.63	6.79	± 3.57	0.399	0.966	0.528

[1] Sum *n*-3 PUFA: C18:3 *n*-3 + C18:4 *n*-3 + C20:3 *n*-3 + C20:5 *n*-3 + C22:5 *n*-3 + C22:6 *n*-3. [2] Sum *n*-6 PUFA: C18:2 *n*-6 + C18:3 *n*-6 + C20:2 *n*-6 + C20:3 *n*-6 + C20:4 *n*-6 + C22:2 *n*-6 + C22:4 *n*-6. [3] Sum PUFA: C18:2 *c*-9, *t*-11 + C18:2 *t*-9, *t*-12 + C20:3 *n*-9 + Sum *n*-3 PUFA + Sum *n*-6 PUFA. [4] Sum MUFA: C14:1 *c*-9 + C16:1 *c*-9 + C17:1 *c*-9 + C18:1 *c*-9 + C18:1 *c*-11 + C18:1 *t*-9 + C18:1 *t*-11 + C20:1 *c*-11 + C22:1 *c*-13. [5] C21:0: includes *t*-10, *c*-12 CLA. [6] Sum SFA: C10:0 + C12:0 +C13:0 + C14:0 + C15:0 + C16:0 + C17:0 + C18:0 + C20:0 + C21:0 + C22:0 + C23:0 + C24:0. NA: Not calculated by SAS software due to small sample size. [a,b,c] Different superscript letters indicate significant differences at $p < 0.05$.

Table 5. Fatty acid concentration (mg/100 g tissue) in kidney fat of calves of four maternal supplementation groups: Control group (CON, n = 8), essential fatty acids (EFA, n = 9), conjugated linoleic acids (CLA, n = 8), EFA + CLA (n = 11). Data are given as LSM ± SE_{LSM}.

Fatty Acid	Supplementation Group								Effect (*p*-Value)		
	CON		EFA		CLA		EFA + CLA		EFA	CLA	EFA * CLA
C18:2 *c*-9, *t*-11	49.92	± 6.30 ^{ab}	35.58	± 5.78 ^{b}	68.13	± 9.07 ^{a}	62.58	± 5.32 ^{a}	0.154	0.002	0.527
C18:3 *n*-3	37.86	± 21.49 ^{c}	120.27	± 19.72 ^{ab}	24.25	± 30.95 ^{ac}	179.10	± 18.15 ^{a}	<.001	0.334	0.133
C18:4 *n*-3	2.48	± 0.27 ^{a}	1.51	± 0.25 ^{ab}	1.58	± 0.39 ^{ab}	1.31	± 0.23 ^{b}	0.044	0.070	0.244
C20:5 *n*-3	3.47	± 0.82 ^{b}	9.08	± 0.73 ^{a}	2.21	± 1.17 ^{b}	10.68	± 0.69 ^{a}	<.001	0.842	0.100
C22:5 *n*-3	24.36	± 2.50 ^{b}	38.88	± 2.30 ^{a}	15.36	± 3.61 ^{b}	45.30	± 2.11 ^{a}	<.001	0.635	0.009
C22:6 *n*-3	13.54	± 2.18	19.78	± 1.92	12.59	± 3.20	20.00	± 1.81	0.008	0.874	0.798
Sum *n*-3 PUFA [1]	84.62	± 23.39 ^{b}	200.15	± 21.46 ^{a}	58.14	± 33.69 ^{b}	270.20	± 19.75 ^{a}	<.001	0.391	0.069
C18:2 *n*-6	571.82	± 56.81	594.53	± 52.12	549.65	± 81.81	663.59	± 47.97	0.274	0.702	0.467
C18:3 *n*-6	12.49	± 1.47	12.14	± 1.34	8.11	± 2.11	10.90	± 1.24	0.448	0.084	0.335
C20:3 *n*-6	112.74	± 12.08	113.85	± 11.08	105.10	± 17.40	117.79	± 10.20	0.600	0.887	0.663
C20:4 *n*-6	113.92	± 8.34	99.50	± 7.65	118.28	± 12.01	107.55	± 7.04	0.173	0.492	0.841
C22:4 *n*-6	45.67	± 4.96	38.90	± 4.55	49.34	± 7.14	44.30	± 4.19	0.279	0.400	0.874
Sum *n*-6 PUFA [2]	884.34	± 71.73	890.02	± 65.81	862.91	± 103.30	978.00	± 60.57	0.441	0.668	0.490
C18:2 *t*-9, *t*-12	5.74	± 0.94	5.31	± 0.83	6.26	± 1.30	6.17	± 0.79	0.786	0.455	0.852
C20:3 *n*-3	2.92	± 1.25 ^{b}	10.66	± 1.15 ^{a}	1.93	± 1.81 ^{b}	13.83	± 1.06 ^{a}	<.001	0.425	0.140
C20:2 *n*-6	27.69	± 4.11	30.23	± 3.63	30.59	± 5.84	33.01	± 3.43	0.566	0.499	0.988
C22:2 *n*-6	0.38	± 0.40 ^{b}	0.78	± 0.37 ^{b}	2.75	± 0.58 ^{a}	0.98	± 0.34 ^{ab}	0.122	0.006	0.019
C20:3 *n*-9	10.64	± 0.92	10.87	± 0.85	10.25	± 1.33	9.14	± 0.78	0.659	0.294	0.509
Sum PUFA [3]	1035	± 88	1142	± 81	1006	± 127	1326	± 75	0.033	0.420	0.277
C14:1 *c*-9	609.73	± 62.79	507.47	± 57.61	491.40	± 90.42	425.66	± 53.02	0.225	0.147	0.792
C16:1 *c*-9	4549	± 415	4998	± 364	4546	± 602	3816	± 343	0.754	0.181	0.178
C17:1 *c*-9	7.37	± 1.88	7.66	± 1.80	7.27	± 2.16	7.70	± 1.77	0.758	0.976	0.950
C18:1 *c*-9	22614	± 957	23365	± 839	22018	± 1374	23662	± 795	0.246	0.880	0.648
C18:1 *c*-11	1341	± 124	1614	± 108	1619	± 182	1472	± 102	0.640	0.609	0.115
C18:1 *t*-9	63.01	± 11.34	54.98	± 10.89	58.17	± 13.02	54.56	± 10.72	0.400	0.691	0.741
C18:1 *t*-11	58.18	± 8.34	44.99	± 7.66	50.03	± 12.02	57.02	± 7.05	0.733	0.829	0.277
C20:1 *c*-11	121.80	± 24.60	147.01	± 22.57	166.61	± 35.42	163.20	± 20.77	0.684	0.256	0.598
C22:1 *c*-13	5.56	± 0.80	5.87	± 0.73	6.75	± 1.15	6.21	± 0.67	0.893	0.376	0.631

C24:1 *c*-15	3.13	± 0.52	3.27	± 0.45	3.31	± 0.75	2.97	± 0.43	0.861	0.913	0.657
Sum MUFA [4]	29499	± 1161	30750	± 1028	28989	± 1621	29717	± 977	0.408	0.504	0.820
C10:0	30.31	± 3.22	26.24	± 2.85	26.50	± 4.50	25.81	± 2.70	0.473	0.509	0.596
C11:0	3.60	± 0.40	3.21	± 0.37	2.94	± 0.58	2.81	± 0.34	0.561	0.230	0.767
C12:0	93.59	± 10.64	58.82	± 9.77	76.81	± 15.33	67.08	± 8.99	0.063	0.711	0.290
C13:0	7.49	± 0.72	5.58	± 0.66	6.01	± 1.03	5.22	± 0.61	0.093	0.243	0.481
C14:0	1802	± 136	1490	± 125	1703	± 196	1519	± 115	0.101	0.810	0.670
C15:0	63.19	± 6.63	48.50	± 5.81	57.89	± 9.75	53.56	± 5.48	0.198	0.987	0.459
C16:0	20678	± 917	19976	± 829	19867	± 1211	20328	± 796	0.886	0.778	0.479
C17:0	499.36	± 29.44	417.29	± 27.01	442.17	± 42.40	448.49	± 24.86	0.243	0.683	0.179
C18:0	6301	± 485	6071	± 442	6591	± 628	6821	± 426	1.000	0.214	0.580
C20:0	56.94	± 7.10	44.38	± 6.29	51.89	± 9.99	53.73	± 5.96	0.468	0.764	0.317
C21:0 [5]	2.49	± 3.11 [b]	2.11	± 2.67 [b]	15.27	± 4.59 [ab]	16.28	± 2.55 [a]	0.927	<.001	0.827
C22:0	3.60	± 0.47	3.51	± 0.44	3.98	± 0.58	3.74	± 0.43	0.660	0.394	0.836
C23:0	14.25	± 2.92	16.65	± 2.60	18.60	± 4.06	15.59	± 2.47	0.919	0.567	0.351
C24:0	4.09	± 0.83	4.03	± 0.74	4.91	± 1.19	4.57	± 0.70	0.822	0.432	0.871
Sum SFA [6]	29546	± 1294	28193	± 1194	28950	± 1632	29329	± 1158	0.649	0.793	0.406
Total fat content (%)	60.32	± 1.87	60.09	± 1.72	59.06	± 2.36	60.46	± 1.67	0.707	0.764	0.587

[1] Sum *n*-3 PUFA: C18:3 *n*-3+ C18:4 *n*-3 + C20:3 *n*-3 + C20:5 *n*-3 + C22:5 *n*-3 + C22:6 *n*-3. [2] Sum *n*-6 PUFA: C18:2 *n*-6 + C18:3 *n*-6 + C20:2 *n*-6 + C20:3 *n*-6 + C20:4 *n*-6 + C22:2 *n*-6 + C22:4 *n*-6. [3] Sum PUFA: C18:2 *c*-9, *t*-11 + C18:2 *t*-9, *t*-12 + C20:3 *n*-9 + Sum *n*-3 PUFA + Sum *n*-6 PUFA. [4] Sum MUFA: C14:1 *c*-9 + C16:1 *c*-9 + C17:1 *c*-9 + C18:1 *c*-9 + C18:1 *c*-11 + C18:1 *t*-9 + C18:1 *t*-11 + C20:1 *c*-11 + C22:1 *c*-13 + C24:1 *c*-15. [5] C21:0: includes *t*-10, *c*-12 CLA. [6] Sum SFA: C10:0 + C11:0 + C12:0 + C13:0 + C14:0 + C15:0 + C16:0 + C17:0 + C18:0 + C20:0 + C21:0 + C22:0 + C23:0 + C24:0. [a,b,c] Different superscript letters indicate significant differences at $p < 0.05$.

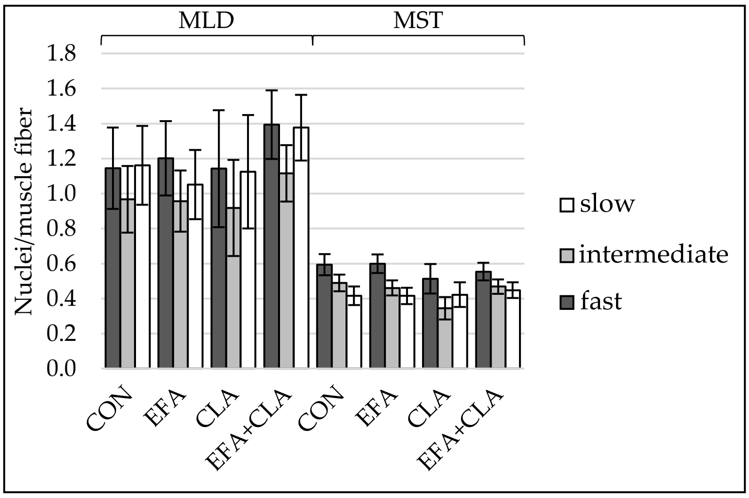

Figure 1. Number of nuclei per muscle fiber of each fiber type in longissimus (MLD) and semitendinosus muscle (MST) of calves of four maternal supplementation groups: Control group (CON, n = 8), essential fatty acids (EFA, n = 9), conjugated linoleic acids (CLA, n = 8), EFA+CLA (n = 11). Data are shown as LSM ± SE_{LSM}.

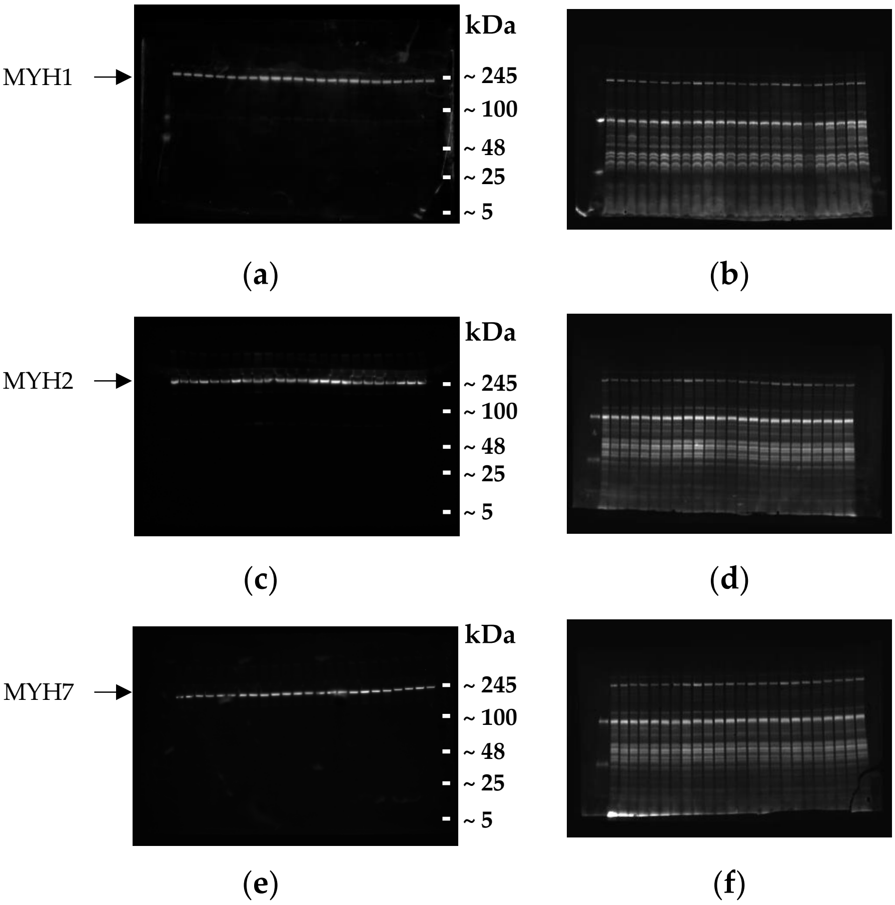

Figure 2. Representative western blot pictures used for protein quantification of myosin heavy chain isoform 1 (MYH1), isoform 2 (MYH2) and isoform 7 (MYH7) in skeletal muscle of calves of four maternal supplementation groups: Control group, essential fatty acids (EFA), conjugated linoleic acids (CLA), EFA+CLA. Blots show target protein (indicated by arrow; a, c, e) or total protein (b, d, f).
